# Functional connectivity changes in meditators and novices during yoga nidra practice

**DOI:** 10.1038/s41598-024-63765-7

**Published:** 2024-06-05

**Authors:** Suruchi Fialoke, Vaibhav Tripathi, Sonika Thakral, Anju Dhawan, Vidur Majahan, Rahul Garg

**Affiliations:** 1 National Resource Center for Value Education in Engineering, Indian Institute of Technology, Delhi, India; 2 Psychological and Brain Sciences, Boston University, Boston, USA; 3 Department of Computer Science, Shaheed Sukhdev College of Business Studies, University of Delhi, Delhi, India; 4 National Drug Dependence Treatment Centre, All India Institute of Medical Sciences (AIIMS), Delhi, India; 5 Mahajan Imaging, Delhi, India; 6 Amar Nath and Shashi Khosla School of Information Technology, Indian Institute of Technology, Delhi, India; 7 Department of Computer Science and Engineering, Indian Institute of Technology, Delhi, India

**Keywords:** Yoga-nidra, fMRI, Default mode network, Meditation, Functional connectivity, Neuroscience, Cognitive neuroscience

## Abstract

Yoga nidra (YN) practice aims to induce a deeply relaxed state akin to sleep while maintaining heightened awareness. Despite the growing interest in its clinical applications, a comprehensive understanding of the underlying neural correlates of the practice of YN remains largely unexplored. In this fMRI investigation, we aim to discover the differences between wakeful resting states and states attained during YN practice. The study included individuals experienced in meditation and/or yogic practices, referred to as ‘meditators’ (n = 30), and novice controls (n = 31). The GLM analysis, based on audio instructions, demonstrated activation related to auditory cues without concurrent default mode network (DMN) deactivation. DMN seed based functional connectivity (FC) analysis revealed significant reductions in connectivity among meditators during YN as compared to controls. We did not find differences between the two groups during the pre and post resting state scans. Moreover, when DMN-FC was compared between the YN state and resting state, meditators showed distinct decoupling, whereas controls showed increased DMN-FC. Finally, participants exhibit a remarkable correlation between reduced DMN connectivity during YN and self-reported hours of cumulative meditation and yoga practice. Together, these results suggest a unique neural modulation of the DMN in meditators during YN which results in being restful yet aware, aligned with their subjective experience of the practice. The study deepens our understanding of the neural mechanisms of YN, revealing distinct DMN connectivity decoupling in meditators and its relationship with meditation and yoga experience. These findings have interdisciplinary implications for neuroscience, psychology, and yogic disciplines.

## Introduction

Yoga nidra (YN) practice, a meditative technique originating from the ancient Indian tradition, has garnered global attention for its potential to improve psychological well-being and health^1^. Despite the growing interest in its clinical applications, a comprehensive understanding of the underlying neural correlates of YN remains largely unexplored^2^. Literally translated as “Yogic Sleep practice,” YN practice typically attempted in *Shavasana*, a supine position resembling the stillness of a corpse which contrasts it from other conventional meditation practices that requires a seated, upright posture thus making it suitable for MRI based scanning approaches. YN practice employs audio-guided instructions which systematically guide the awareness of the participant to different parts of the body, breathing, or *mantras*^1^ that aim to induce a deeply relaxed state, mirroring the serenity experienced during deep sleep but with conscious awareness in contrast to the self-regulated focus typically associated with focussed attention style of meditations. The practitioner remains in a state of light withdrawal of the five senses (*pratyahara*)^3^ with four of their senses internalized and only the hearing still connects to the instructions. The exceptional allure of this technique stems not merely from the deep relaxation and mindful awareness it provides, but also as a method to progressively master entering the most profound states of meditation (*samādhi*)^3–5^.

YN is an easy to practice and accessible meditation technique that has demonstrated impact on physiological, psychological, and cognitive improvements, making it a promising technique for clinical interventions^2,6^. Specific examples include improvement in symptoms of stress, depression, and anxiety^7–11^, migraine^12^, pain^13^, menstrual abnormalities^14,15^, somatoform disorders^16^, diabetes^17^, and hypertension^10,18^. YN practice has also shown benefits in improving the outcomes related to insomnia and sleep quality in several clinical studies^19–22^. YN is adaptable to different traditions and customs and a variant of YN known as iRest has been specifically designed to treat post traumatic stress disorder (PTSD) and the sleep quality of U.S. war veterans^23,24^. Custom iRest protocols are applied to several other therapeutic contexts including occupational stress, sexual trauma, and insomnia^25–27^. Some recent protocols like the non-sleep deep rest (NSDR) which utilize YN have expanded its reach and acceptance^28^.

Multiple studies have looked at the EEG signatures of YN. Parker et. al. hypothesized that the ‘perfect’ state of YN would be akin to deep non-REM sleep (exclusive delta-wave predominance) with maintained internal and external awareness, finally culminating into *turīya* (a state devoid of phenomenological content)^29^. Non-peer-reviewed reports of pilot studies have claimed that accomplished yogis have demonstrated such states during YN^5,30^. Two pioneering PET-EEG studies reported widespread increases in theta activity during YN (compared to a resting state), indicating a shift towards a more relaxed state^31,32^. The first PET-EEG study with highly experienced yoga practitioners (n = 9) revealed bilateral hippocampal activation, along with activation in Wernicke’s area, the occipital lobe, and the parietal and frontal lobes during different stages of YN. Subjectively, the participants described the YN state as “reduced conscious control of attention and behavior, relaxation and a loss of will”, distinctly different from a control condition of resting state^31^. The second PET-EEG study involving eight experienced yoga teachers, revealed a 65% increase in dopamine release during YN. This increase was associated with decreased activity in the striatum, as well as reduced executive control and attentional engagement^32^. Interestingly, a clinical EEG study revealed that compared to the resting state pre-YN, there was an increase in delta power (and no change in theta power) in the central area and a decrease in delta power in the prefrontal area during YN. These findings were attributed to local sleep and not regular sleep during the practice of YN^19^. In a separate EEG study, six novices underwent 12 YN sessions, reporting altered states of consciousness according to the phenomenology of consciousness inventory. Intriguingly, despite each 2 h YN session, no EEG recordings from any subject showed sleep indicators like K-complexes or sleep spindles^33^.

Although the number of neuroimaging studies on YN is limited and heterogeneous, consisting only of two PET studies and no fMRI study to our knowledge, they provide valuable insights into the state of mind during the YN practice. Along with the EEG studies, these studies have revealed changes indicative of sleep-like relaxation, altered consciousness, and modulation of dopamine release. Despite its recognition in wellness and theoretical consciousness studies, it has received limited attention in terms of its neural underpinnings. Existing research and theoretical postulations suggest that YN practice might influence specific neural circuits involved in mind-wandering, self-regulation, and attention. However, these interpretations are mostly inferred from broader meditation research^34^, and the unique characteristics of YN—its blend of sleep-like relaxation with conscious awareness—remain underexplored. This gap in knowledge forms the foundation for our study as we seek to illuminate the neural correlates of YN.

In the context of investigating the neural correlates of YN, the default mode network (DMN) assumes specific importance given its involvement in higher cognition such as internal mentation^35^, self-projection^36^, theory of mind^37^, intrinsic processing^38^, and mind wandering^39^, It lies at the further end of the hierarchy of cognitive networks and evolved when the neocortex expanded^40^. The DMN deactivates during externally demanding tasks in an anti-correlated manner with the dorsal attention network (DAN)^41^. DMN is involved in scene construction which activates when the brain projects itself into the past or the future or is engaged in thinking about others^42^. Alpha and beta wave activity is closely linked with DMN activations^43,44^, and could represent an integrated system. The dysfunction of DMN is associated with various mental disorders like Alzheimer’s disease^45^, anxiety, depression, and ADHD^46^.

Meditative practices affect the activity in the DMN, especially in the anterior cingulate cortex (ACC)^47^, and medial prefrontal cortex (mPFC)^48^, and are associated with anxiety relief^47^, emotional stability^49^, and reduced mind wandering^50^. Mindfulness practices have demonstrated that around six to eight weeks of mindfulness training results in changes of functional connectivity between the DMN and hippocampus^51^, between DMN-DAN and DMN-visual cortex^52^, between DMN and Salience network^53–55^ and within DMN^56,57^ pointing towards large scale brain reorganization with meditation usually centered in the DMN. Long term practitioners in Vipassana and other Theravadan Buddhist traditions have shown DMN centric brain changes with increased anticorrelations with DAN^58^ and modified connectivity within DMN regions and increased coupling with the central executive networks^59^.

In this study, we used fMRI to explore the neural correlates of YN practice during a 20 min practice of YN in a group of meditators (n = 30) and a matched group of novices (n = 31). We specifically compare the effects of the YN practice with resting states recorded both before and after the practice of YN. Our investigation delves into how YN practice impacts the DMN’s functional connectivity, and how these changes correlate with the total duration of an individual's meditation practice. More specifically, in this paper, we aim to find out: does the functional connectivity within the brain, as captured through seed regions of the DMN during YN practice, resemble the patterns seen during sleep or is it similar to a wakeful rest? And are there differences in DMN interconnectivity between regular meditation practitioners and novices during YN practice and resting state sessions?

## Results

**Figure 1:**
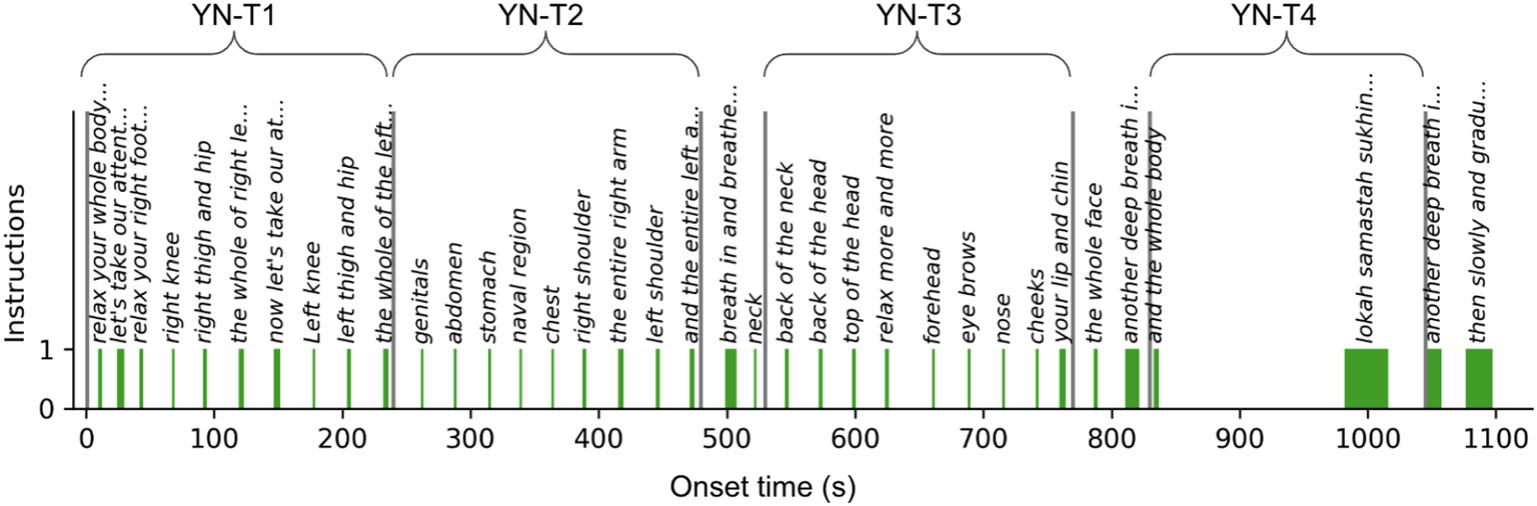
Illustration audio-guided paradigm of the yoga nidra practice. The x-axis represents the progression of subtitles over time, while the y-axis denotes the presence of the audio instructions. The width of the bar represents the duration of the instruction. The practitioner’s attention is sequentially guided to different body parts as depicted in the figure. The paradigm is divided into four periods for connectivity analysis: T1 to T3 each lasting 240 s, and T4 spanning 215 s chosen to exclude any explicit breathing instructions.

We gathered fMRI and subjective questionnaire data from meditation practitioners (n = 30) and demographically matched novice controls (n = 31); see Table 1 for the demographics details.Query We analyzed the audio-guided yoga nidra (YN) paradigm (in English in the voice of Sri Sri Ravi Shankar, founder Art of Living) and pre- and post-YN resting state scans. The YN script used in the fMRI paradigm is represented in Fig. 1. The paradigm is divided into four periods: T1 to T3 each lasting 240 s, and T4 spanning 215 s for functional connectivity (FC) analysis. The instructions systematically guide the practitioner’s attention across different body parts followed by a mantra chant and period of silence, thereby fostering a relaxed state.

**Table 1:** Participant characteristics.

Demographics variable	Meditators	Controls	p-value
No. of subjects	30	31	
Subjects excluded based on low auditory activations	2	7	
Included subjects	28	24	
Sex			
Count of Females	4	4	0.812
Marital status			
Count of Married	4	5	0.534
Age (years)			
Mean ± std	27.57 ± 3.53	25.96 ± 3.50	0.105
Body weight (kg)			
Mean ± std	67.57 ± 14.10	66.52 ± 13.58	0.789
Formal education (years)			
Mean ± std	17.00 ± 1.59	17.08 ± 2.02	0.868
Family size (No of members)			
Median	4	4	
Overall regular practice [asana+pranayama+meditation] (hrs)			
Mean ± std	2516.98 ± 1839.21	21.50 ± 28.92	0.000

### BOLD activations modeled after YN auditory instructions

We performed the general linear model (GLM) analysis with the aim to model the BOLD response in relation to the audio instructions. Accordingly, the explanatory variable (EV) in the model was the presence of audio instructions, coded as 1 for the duration of an auditory instruction (Fig. 1).

As described in the methods section, subjects who didn’t comprehend auditory instructions were identified from GLM analysis based on their FDR corrected activation maps. This led to the exclusion of two out of 30 meditators and seven out of 31 controls from subsequent analysis.

**Figure 2:**
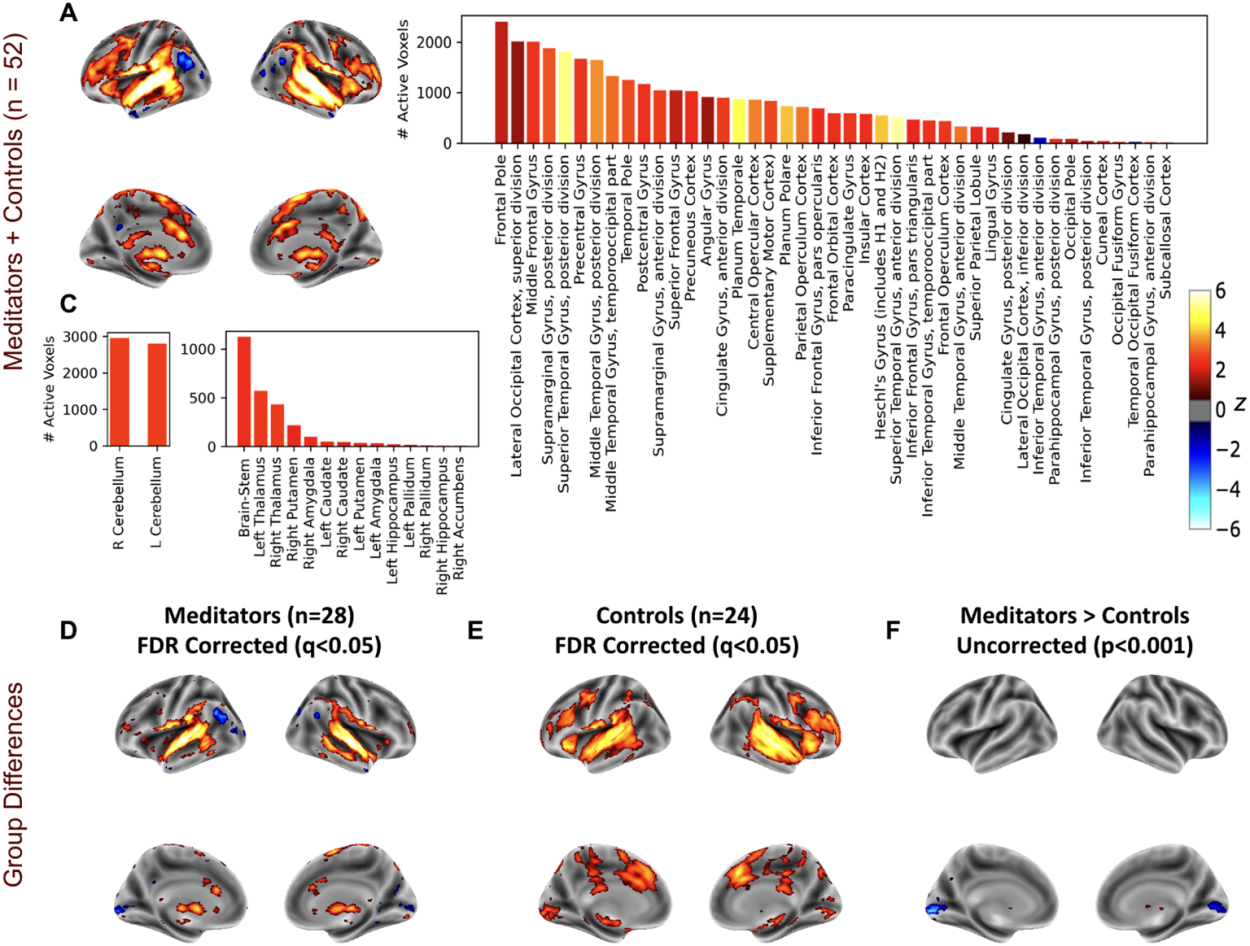
Brain activations during yoga nidra, show stimulated auditory, language, motor, and limbic regions, and distinctive thalamic activation in meditators. (**A**) Surface visualizations of z-statistics, adjusted using false discovery rate (FDR) correction (q < 0.05), derived from GLM analysis of yoga nidra, all subjects (meditators and controls collectively). The explanatory variable (EV) was modeled with the presence of any audio instruction as one (Fig. 1). (**B**) Active voxel counts for cortical ROIs from the above map are presented (the color of the bars represents the average z-score within that ROI). Consistent with the auditory instructions involved in yoga nidra (YN), which guide attention to different body parts, activations were observed not only in the auditory and language regions but also in motor, supplementary motor, and somatosensory areas. Regions associated with memory and emotional processing are also active including Insula and parahippocampal gyrus. Interestingly, despite activation of language and auditory areas, DMN regions do not exhibit concurrent de-activation. (**C**) Active voxel counts for cerebellum and subcortical ROIs exhibiting activations in limbic regions including the brainstem, thalamus, and amygdala (the color of the bars represents the average z-score within that ROI). (**D**) Surface plots of z-stats, FDR-corrected (q < 0.05), from GLM analysis of yoga nidra for meditators (n = 28) and (**E**) same for control subjects (n = 24). (**F**) Although the FDR-corrected maps (q < 0.05) from the comparison ‘meditators > controls’ didn’t have significant clusters, uncorrected maps with a threshold at p < 0.001 underscore bilateral activation in the thalamic regions and deactivation in bilateral occipital pole for meditators.

Group-level activations for meditators (n = 28) and controls (n = 24) observed during the practice of YN based on GLM analysis are presented in Fig. 2. Additionally, in Table S2, we provide a list of significant clusters of activations and deactivations. YN practice initiates activations in multiple brain regions specifically, the temporal gyri, responsible for auditory and language processing, are activated, highlighting the key role of auditory reception and comprehension during YN practice. The supplementary motor area (SMA) and the postcentral gyrus (PCG) regions that are involved in motor planning and control are activated as expected as the instructions involve scanning of body parts. Other areas include the thalamus and brainstem, crucial for the relay of sensory and motor signals^60^. Activations in the anterior cingulate cortex (ACC) well-known for its role in both cognition and emotion is notable^61^. Similarly, activation in the insula, inferior frontal regions, cerebellum, and brainstem, key regions involved in emotional processing and cognitive control were observed. Lastly, superior frontal gyrus, contributing to high-level cognitive functions, also exhibit activation. However, deactivation in several regions, including the frontal pole, superior frontal gyrus, and other areas may suggest a decreased demand for some complex cognitive processes, reinforcing the relaxing and introspective nature of YN. Figure 2D–F offer a comparative view of the BOLD response to YN instructions in meditators versus controls. Thalamic activation was observed in meditators but not in the control group. Remarkably, despite the activation of language, and auditory areas, as participants attended to the instructions, DMN regions did not exhibit concurrent de-activation.

### Phenomenological self-reports of YN practice

Both groups were instructed to practice YN (same script as the one used during fMRI) 2–3 times in the weeks prior the scanning day. Most meditators (15/28) and controls (17/24) indicated practicing 1–5 times in the past month with two controls and four meditators reported practicing 6–10 times and three meditators reported practicing > 10 times in the past month. Five controls and five meditators reported no practice in the past month and remaining didn’t respond. We also collected the subjects’ subjective experience during the scan, including the number of times they think they fell asleep, and restfulness. The survey results indicated, most control participants (19/24) reported falling asleep < two times, with a smaller number (2/24) falling asleep 3–4 times, and two participants experienced constant falling in and out of sleep. Similarly, in the meditators group, a majority (24/28) reported falling asleep < two times, one fell asleep 3–4 times, three reported constantly falling in and out of sleep. The remaining subjects did not respond to this question. Despite some of these subjects reporting falling asleep at least for some time during the scan, they had significant (FDR corrected) activation in the language comprehension areas indicating that they were attentive and comprehended the audio instructions during the scan.

### DMN connectivity during YN

#### Group DMN FC during YN and resting states

For the seed based FC analysis, we selected the following seeds within the default mode network (DMN): the posterior cingulate cortex (PCC), medial prefrontal cortex (mPFC), right inferior parietal lobule (right-IPL), and left inferior parietal lobule (left-IPL)^62^. We extracted temporal sequences from a 10 mm-radius sphere centered on these seeds, and computed pairwise correlations with all brain voxels, followed by Fisher’s Z-transformation for group-level analysis. The outcomes are displayed on surface maps in Figs. S1–S4 for all four DMN seeds in both meditators and controls. The presence of a functional DMN manifested during both resting states and YN. Notably, discernible patterns of FC emerged between meditators and controls during the YN practice for each DMN seed. This divergence prompted an exploration of contrasts between the two groups and the stages of YN practice versus resting states discussed in the subsequent sections.

#### Comparison of the DMN FC in meditators and novices

**Figure 3:**
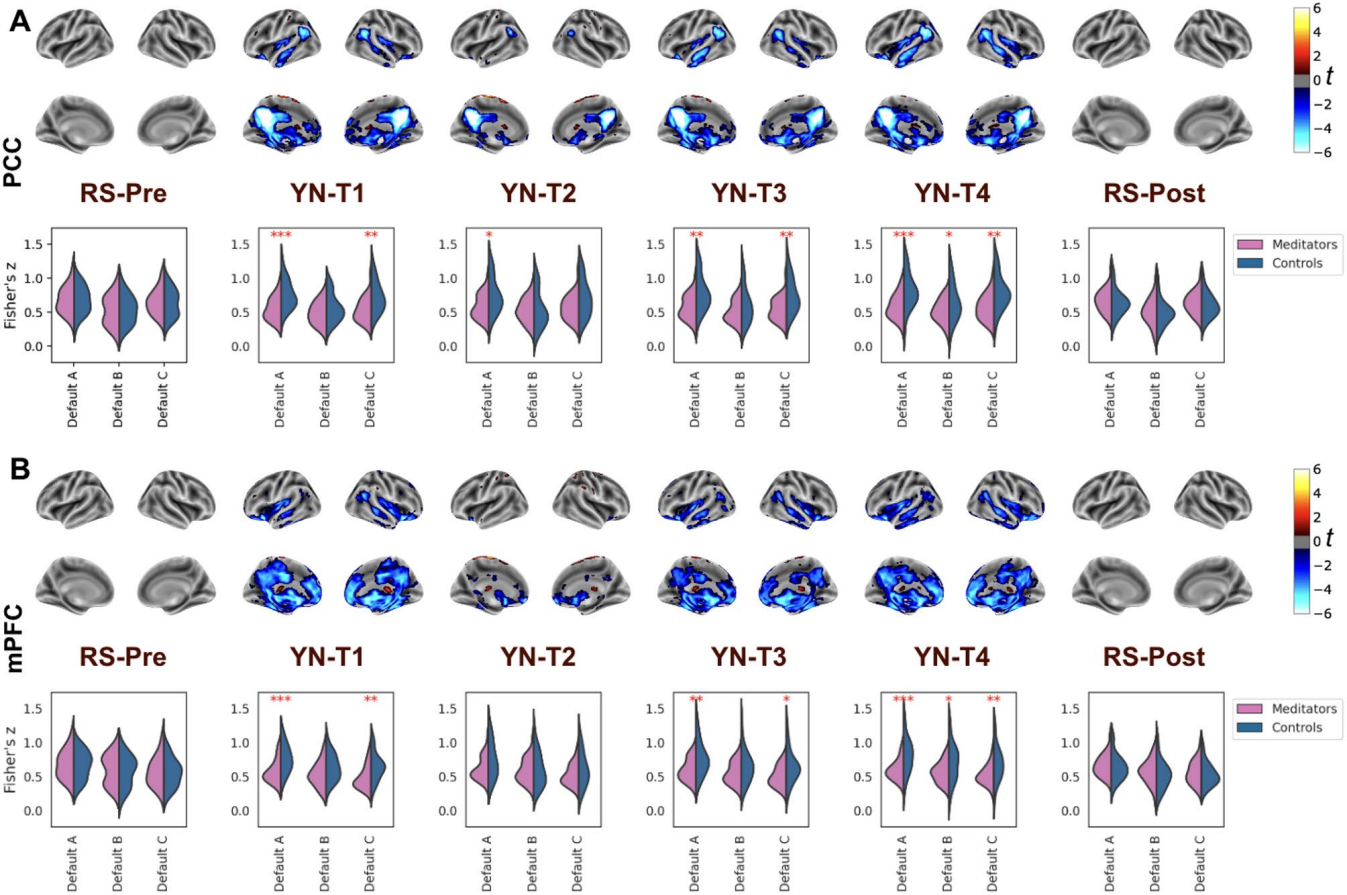
Group differences in DMN-FC between meditators and controls during yoga nidra but not during resting states. The figure uses two-sided *t*-tests to compare FC in meditators (n = 28) and controls (n = 24) using DMN seeds (**A**) posterior cingulate cortex (PCC) and (B) medial Prefrontal Cortex (mPFC). The surface plots display the t-values, corrected for multiple comparisons (FDR-corrected, q < 0.05), across the four stages of Yoga Nidra (YN), as well as in resting states pre and post-YN. The accompanying violin plots illustrate the distribution of Fisher’s z-values for both groups during these stages. These plots represent the averaged Fisher’s z-values of the seeds within three distinct default mode network (DMN) subdivisions (default A, default B, and default C), as outlined by the Schaefer cortical Atlas. The width of the violin plot at any given y-value (Fisher’s z-value) represents the proportion of data located there, providing a visual representation of the data's distribution (* represents statistical significance with p < 0.05, ** with p < 0.01 and *** with p < 0.001). Notably, meditators demonstrate significantly reduced DMN connectivity for both PCC and mPFC seeds during all four stages of yoga nidra, indicating a deeper state of focused relaxation in experienced meditators. Conversely, no significant differences were observed in the FC of these seeds in either of the resting states.

Figure 3 suggests differences in the FC patterns between meditators and non-meditators during YN. Two-way ANOVA analysis across DMN seeds (PCC, mPFC, right-IPL, left-IPL) to Default A network, revealed significant group effects (F range: 17.419–30.005, all p < 0.001; Table S7), with no significant main effects of stage (F range: 0.287–0.599, all p > 0.05; Table S7). Specifically, during all four stages of YN, meditators exhibit markedly reduced FC with the four DMN seeds, PCC (Fig. 3A, Table S3), mPFC (Fig. 3B, Table S4), right-IPL (Fig. S5A, Table S5), and left-IPL (Fig. S5B, Table S6). Interestingly, this pattern of reduced DMN connectivity emerges in the first phase, T1 itself (all seeds -> Default A, p < 0.05, Tables S3–S6), which lasts only four minutes suggesting that the reduced FC within the DMN seen in meditators is indicative of a state of deep relaxation and not due to sleep. The effect is strongest in the last stage of YN, T4 (all seeds -> Default A, p < 0.01, Tables S3–S6). Moreover, no significant post-hoc differences were detected in the FC of these DMN seeds between meditators and non-meditators during resting states before or after YN (all seeds -> Default A, p > 0.05, Tables S3–S6). To rule out effects of head motion or physiological differences on YN FC results, we found no significant differences in framewise displacement (Fig. S12) or in heart and breathing rates between meditators and controls (Fig. S11).

#### DMN during YN versus during resting state

**Figure 4:**
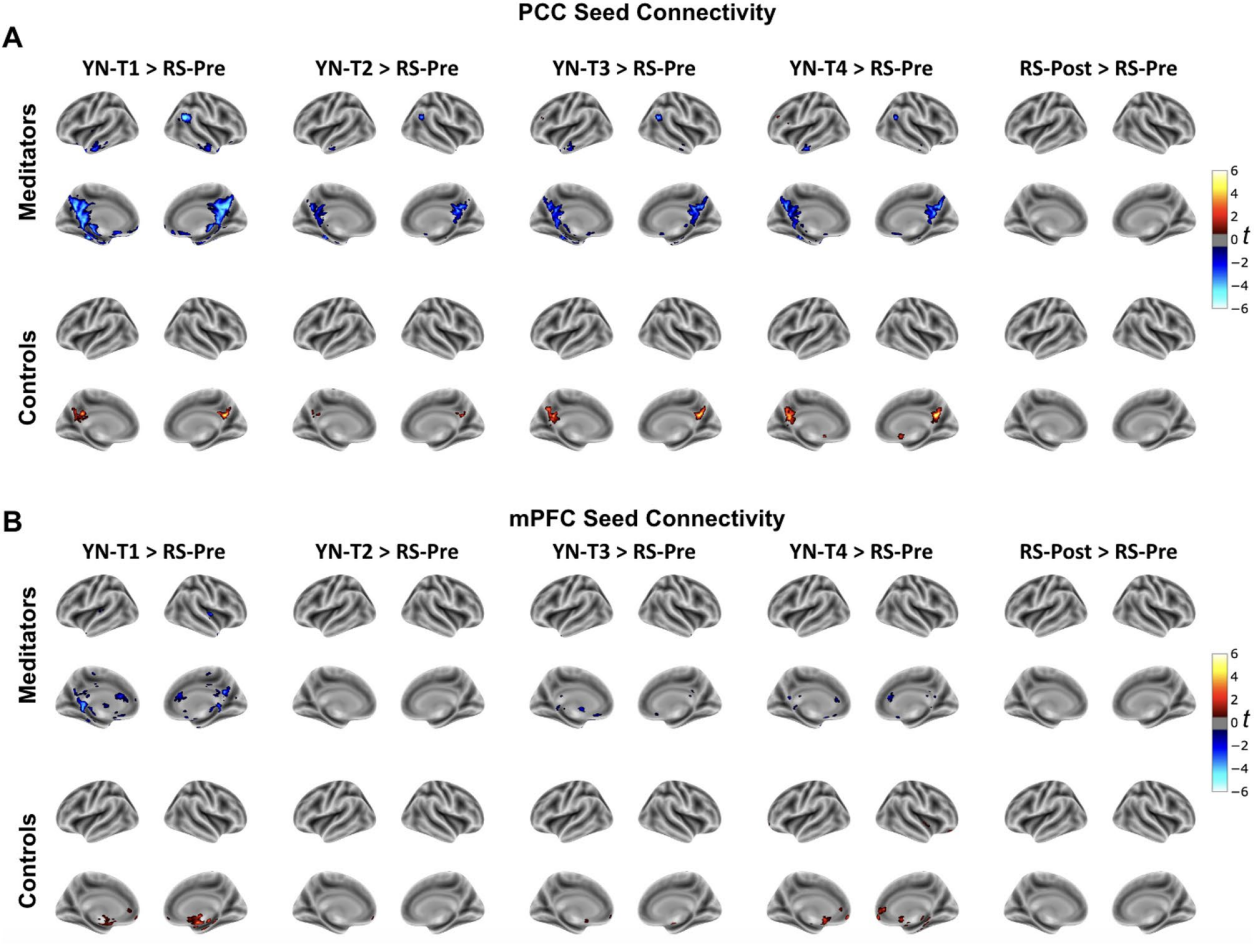
Different functional connectivity changes emerge in meditators and controls during yoga nidra compared to rest. For (**A**) PCC and (**B**) mPFC seeds, a comparison of the FC during YN (T1 through T4) and the resting state post-completion of yoga nidra (RS-Post) is performed with resting state pre-yoga nidra (RS-Pre) as a baseline. The surface maps present t-values from two-sided *t*-tests, corrected for multiple comparisons (FDR-corrected, q < 0.05), providing a visual representation of FC differences. The figure highlights that meditators demonstrate a significant decrease in DMN connectivity during YN compared to their resting state. Conversely, controls display an increase or no change in connectivity during YN relative to the resting state.

The ANOVA analysis across DMN seeds (PCC, mPFC, right-IPL) to Default A network, revealed significant group by stage interactions (F range: 2.711–4.054, all p < 0.05; Table S7). In the context of both meditators and controls, we further contrasted the FC during stages of YN with the FC during the resting state prior to YN (RS_Pre). For PCC (Fig. 4A), mPFC (Fig. 4B), right-IPL (Fig. S6A), and left-IPL (Fig. S6B) seeds, the findings are divergent between the two groups. Meditators exhibit a DMN FC reduction throughout YN (T1–T4) compared to their resting state. Contrarily, control subjects display a slight increase or no change in DMN FC during YN compared to their resting state. In the SI (Fig. S7), we also include the intra-DMN FC by looking at the correlation between the DMN seed pairs. It is interesting to note that the PCC-mPFC FC is significantly reduced during YN compared to the resting state for meditators (T1 & T3, t-range: − 2.78– − 2.03, p < 0.05) but not for controls (all stages, t-range: − 0.33–0.49, p > 0.05). However, for both meditators and controls, the resting state post YN, wasn’t significantly different (all seed pairs, all stages of YN, p > 0.05) from the resting state prior to YN.

#### Relationship between total meditation practice and DMN-FC

**Figure 5:**
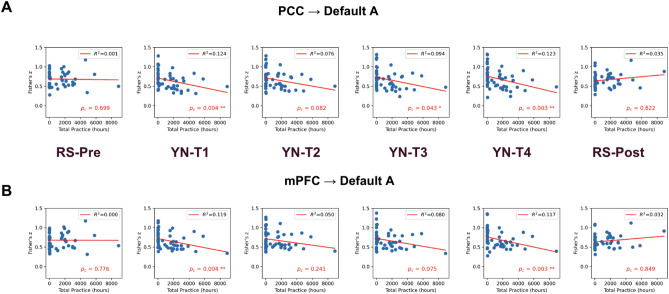
Increased meditation experience significantly correlates with reduced functional connectivity between the mPFC seed and DMN regions. A significant negative correlation between the total duration of meditation practice and the FC of the (**A**) PCC and (**B**) mPFC with other regions of the default mode network (DMN, Default A as defined by the Schaefer Atlas) during the yoga nidra (YN) practice. * Represents statistical significance with p_c_ < 0.05 and ** with p_c_ < 0.01; the p-values are obtained via Spearman ranked test and FDR corrected for multiple comparisons (Table S1). This pattern suggests that increased meditation experience correlates with reduced FC between the mPFC seed and DMN regions during YN, potentially reflecting a trait-like effect of diminished self-referential thought processes during this meditative state. Interestingly, no such linear relationship was detected during the resting states before and after YN, emphasizing the context-dependent nature of this association.

Self-reported cumulative practice hours include yoga aasana, pranayama and meditation are available for all subjects (see Table 1 for details). Figure 5A highlights a compelling negative correlation between the total duration of meditation practice and the FC of the PCC seed with core regions of the DMN (Default A as defined by the Schaefer Atlas^63^) during the YN practice (YN-T1: r(50) =  − 0.352; p = 0.004; YN-T2: r(50) =  − 0.276; p = 0.082; YN-T3: r(50) =  − 0.307; p = 0.043, p = 0.010; YN-T4: r(50) =  − 0.350; p = 0.003). This intriguing pattern suggests that as meditation experience increases, there is a corresponding decrease in FC between the PCC seed and DMN regions during YN. The relationship of meditation experience with mPFC (Fig. 5B), and bilateral IPL seeds (Fig. S9) also reveals similar trends, although their corresponding p-values were slightly higher (Table S1). For both RS-Pre and RS-POS no significant trend was observed for any seed (all p-value > 0.05, Table S1). To ensure the robustness of our findings, we repeated the analysis after removing the outlier subject with more than 8000 h of cumulative practice (Figs. S8, S9, Table S1) and reveal similar results. For the last stage of YN, stage T4, correlation results (Table S1) were strongest across all seeds (corrected p-value range 0.003–0.005) without outlier (corrected p-value range 0.006–0.011).

## Discussion

Yoga nidra (YN), a unique practice combining detailed body scanning with deep relaxation and heightened awareness, is subjected to fMRI scrutiny for the first time in our research. This investigation involves both regular meditators and controls, aiming to uncover the specific neural activations and connections at play during YN practice. Central to our inquiry is determining its distinction from wakeful rest states and comparison between meditators and controls. Resting state scans conducted before and after YN sessions provide suitable control conditions. Simultaneously, the wealth of existing research on functional connectivity (FC) of DMN during various sleep stages offers a comparative landscape.

GLM analysis modeled after presence of audio instruction, revealed activations in regions involved in auditory and language comprehension, as well as motor planning in both groups. Furthermore, we note activations in areas associated with emotional regulation including the ACC, insula, and the limbic system. This observation aligns with the Swami Satyananda Saraswati’s proposition that “Yoga nidra develops control of the emotional reactions and autonomic responses”^1^.

Our analysis additionally uncovered an intriguing distinction between meditators and controls—meditators exhibit pronounced bilateral thalamic activation, a response absent in controls. This finding gains significance when juxtaposed with research that delved into the neural response to auditory stimuli during various stages of sleep employing fMRI and EEG recordings^64^. The study unveiled bilateral activation in the auditory cortex, caudate, and thalamus, during wakefulness and nonrapid eye movement (NREM) sleep. However, they noted a marked reduction in the activation of the thalami during NREM sleep compared to wakefulness^64^. The thalamus, often considered the ‘gateway to consciousness’, plays a pivotal role in relaying sensory information and maintaining arousal^60,65^. Therefore, its increased activation in meditators may reflect a heightened state of awareness, even amidst the deep relaxation characteristic of YN.

The GLM analysis, however, revealed no deactivation in DMN regions, despite concurrent activation in areas associated with the auditory instructions (Fig. 2). There could be three explanations for this. First, YN practice could inherently differ from typical attention-demanding tasks, i.e. the guided and calming nature of YN might foster a degree of engagement that supports the maintenance of DMN connectivity. The absence of DMN deactivation during YN in our study suggests a unique state of consciousness distinct from traditional focused attention (FA) meditation^34,66^. Second, it could indicate a reduction in mind wandering/autobiographical processes during the YN practice^67^. Alternatively, it is possible that disengagement of the DMN regions following the audio instruction might not be strictly stimulus-locked and could exhibit significant variability both across instructions and between subjects. A recent study reported diminished stimulus-locked synchronization in DMN regions in a variety of tasks^68^.

In meditators, significantly reduced FC was observed for each DMN seed (PCC, mPFC, right-IPL, left-IPL) compared to novices at every stage of YN. The body of research comparing the DMN FC of meditators and control groups during meditation is sparse, and notably, no studies have specifically investigated YN. Particularly, Brewer et al. revealed that compared to controls, expert meditators showed enhanced connectivity between the PCC seed and ‘task-positive’ areas (dACC and dlPFC) across three different meditation types as well as during resting state. This increased connectivity with the dACC and dlPFC was not seen using the mPFC as the seed^67^. The difference between meditators and novices during YN may be due to the unique nature of YN which uses guided instructions to evoke a deep relaxation akin to profound sleep, unlike traditional meditation’s wakeful nature. In a seminal review and meta-analysis of fMRI meditation studies, Fox et al.^34^ analyzed common styles of meditation as focused attention, mantra recitation, open monitoring, and compassion/loving-kindness and suggested differences for three others (visualization, sense-withdrawal, and non-dual awareness practices) while keeping YN in a different but unexplored category^34^. Studies on expert meditators have found both state and trait level changes in the activity and connectivity of the various regions of the DMN^69,70^. Meditative states are associated with reduced activity in DMN as compared to resting state^59,67,70,71^. Experienced practitioners have reduced FC between DMN nodes as compared to controls during meditation^67,71,72^ and even at rest^50,73,74^. But some studies have shown increased within-DMN connectivity during meditation especially with mPFC region^72,75^ highlighting some discrepancies in the field possibly due to heterogeneity in meditation styles. DMN connectivity with other networks like the salience and frontoparietal control network change during meditation^55,58,76^.

Interestingly, no significant difference between meditators and novices was noted in the resting states either pre or post-YN. Several studies on the resting state have shown that prolonged and regular meditation practitioners have altered DMN FC compared to novices^67,69,77^. Whereas, some have found no significant differences in DMN FC between meditators and controls during rest^78^. We conjecture that the alteration in traits may necessitate a more extended period of practice and could also be influenced by the demographics and lifestyle of the study population and the specific type of meditation practiced. Notably, yoga practitioners involved in our research are regular householders with an average of 2517 ± 1839 h of yoga practice (which includes yoga postures, breathing, meditation), whereas previous studies documenting changes in resting state FC predominantly focus on meditators (usually renunciate monks) with an excess of 10,000 h of meditation practice.

When comparing any stage of YN with the resting state, meditators demonstrate a decoupling in DMN connectivity with several brain regions, whereas controls display an increased coupling of the DMN. These divergent patterns potentially reflect the ability of experienced meditators to regulate their mind and brain states during deep stages of relaxation, in contrast to non-meditators who perhaps experienced boredom, discomfort, or increased mind-wandering. In prior studies, experienced meditators have shown to have reduced deactivations in DMN during meditation^49,67^ and overall modified functional connectivity of DMN nodes^58,59,66,79^ highlighting that meditators demonstrate reduced mind wandering episodes during both task paradigms and meditation.

Simultaneous EEG-fMRI studies which have investigated sleep depth effects by analyzing EEG features such as theta activity (4–8 Hz) or slow-wave activity (SWA) (< 4.5 Hz)—reveal sustained DMN FC during light sleep, but this connectivity, inclusive of DMN, disintegrates during deep sleep^80,81^. Nevertheless, given the evident reduction in FC of meditators during the initial phase T1, attaining a veritable state of deep sleep appears improbable. This conclusion finds reinforcement in the phenomenological accounts of the meditators, with the majority reporting not experiencing sleep during the practice. This observed decline in FC of meditators underscores the potential of YN practice in modulating the DMN, shifting the brain from a potentially distractible, mind-wandering state to an awake yet deep-sleep like relaxed state.

Remarkably, a significant correlation is observed between the total duration of meditation practice and a reduction in FC of DMN seeds with DMN regions during YN. As illustrated in Fig. 5, we present scatter plots that vividly depict this relationship, with DMN seeds’ FC, averaged within DMN regions, plotted against the accumulated hours of meditation practice. Notably, these alterations appear to predominantly impact the connectivity between the PCC and mPFC seeds and DMN regions. Given that the mPFC is associated with self-referential thought processes^82^, this reduced connectivity might be indicative of reduced self-related thinking or mind-wandering during YN among experienced meditators. For the bilateral IPL seeds, a similar trend was noted but with less statistical significance. This relationship, again, is not observed during resting states, highlighting the context-dependent nature of these meditation-related changes in brain function. Together, these results seem to provide evidence of a trait-like effect of meditation. As one’s experience in meditative practices grows, we observe discernible changes in the FC of key DMN regions during the practice of meditation. Such insights highlight the transformative power of regular meditation practices, especially when combined with techniques like YN.

This study opens up new avenues for future research, for instance, in exploring pathways associated with YN’s efficacy in contexts of neuropsychiatric disorders linked to hyperconnected DMN function^83,84^. When dealing with clinical populations, it’s common to discover that various meditation/yoga techniques have varying degrees of effectiveness for specific disorders and hence, effectively choosing the most suitable practice for an individual patient is a crucial step^85^. The results of this study can provide valuable insights for guiding mental health interventions and enhancing our understanding of the mechanisms underpinning ongoing clinical investigations. Several clinical studies already indicate that the YN practice may mitigate anxiety, stress and depression^8–10,86^ and many have shown promising results of iRest for the same^25,27,87,88^. Also, the promising results of iRest in PTSD, demonstrated by many studies^23,26^, offer compelling evidence to support the belief that YN should prove beneficial for the same. Further research into the mechanisms underlying YN’s impact on neuropsychiatric disorders, mental health and well-being is warranted and holds significant promise.

### Limitations and future directions

One limitation of our study is the standalone use of fMRI, which, while providing detailed spatial information, lacks the temporal resolution of EEG. This may have affected our ability to capture rapid changes in neuronal firing and synchrony during YN. Future studies could enhance our understanding of YN by incorporating simultaneous EEG and fMRI recordings. This would allow for a more detailed examination of the spatiotemporal dynamics across various stages of YN and their distinction from different phases of sleep. While we used unstructured questionnaires to evaluate the phenomenological experience of the subjects during YN, future fMRI research on YN could benefit from the inclusion of validated questionnaires on mindfulness such the mindful attention and awareness scale^89^ or the Toronto mindfulness scale^90^. Furthermore, incorporating a longitudinal study design is critical to better understand the temporal effects and long-term implications of YN on brain activity. This design will enable us to monitor changes over time and elucidate whether regular practice induces any structural or functional brain changes, providing a deeper understanding of the enduring impacts of YN. It is worth noting that both the meditators and controls groups in our study had only 16% females, which could be addressed in future research to ensure gender diversity.

## Conclusion

In this study, we underscore several important insights into the neural underpinnings of yoga nidra (YN), a unique yogic practice. GLM analysis, which was modeled using the presence of audio instructions, revealed no deactivation in DMN regions, despite concurrent activation in areas associated with the auditory instructions. Further, our findings highlight a significant reduction in DMN FC among meditators compared to novices across all stages of YN. Parallelly, the GLM modeling of auditory stimuli produced higher activation in bilateral thalamus for meditators compared to novices. This suggests a unique neural adaptation in meditators during YN which results in being restful yet aware. Interestingly, no such difference in DMN FC was observed between meditators and novices during resting states, either pre or post-YN. Further, when juxtaposing any stage of YN with the resting state, we observed a decoupling in DMN connectivity among meditators, in stark contrast to controls who displayed an increase. This divergence potentially mirrors the advanced ability of meditators to regulate their mind and brain states during deep stages of relaxation, setting them apart from non-meditators. Finally, our analysis reveals a crucial finding: a significant correlation between the total duration of meditation practice and a reduction in FC of DMN seeds to DMN regions during YN. This suggests that sustained yoga practice may lead to enduring changes in the FC of DMN. Our findings set the stage for future research to delve deeper into the cognitive and perceptual correlates of YN, particularly how its practice influences DMN connectivity in a context-dependent manner. Moreover, this research holds significant interdisciplinary implications, bridging the gap between neuroscience, psychology, and the study of yogic practices. The insights gained from this study could potentially inform therapeutic interventions in mental health, providing a scientific basis for the integration of YN in stress management and cognitive therapy programs.

## Materials and methods

### Participant recruitment and experimental design

The study engaged local meditation practitioners in Delhi’s National Capital Region (NCR), utilizing community contacts for recruitment. Participants were classified into two groups: meditators with at least 1 year of regular yoga or meditation experience, and controls, novice-meditators or non-meditators, age and gender-matched with meditators, with less than 6 months of yoga or meditation experience (see Table S1 for demographics). In this context, the term “meditators” pertains to subjects with at least 1 year of regular practice that included asana (yoga postures), pranayama (breathing practices), and meditation. Criteria for inclusion encompassed ages 18–60, a minimum of 10th standard education, English proficiency, and willingness to provide informed consent. Exclusion criteria encompassed diagnosed medical illnesses or on any prescription medication, or unwillingness to sign the informed consent form. The study was approved by the Institutional Review Board at Indian Institute of Technology, Delhi.

The overall study paradigm included two guided meditations: a 20 min yoga nidra (YN) protocol and a 23-min body scan protocol. Each meditation had a 5-min eyes-closed resting state run before and after. We also included a paced breathing run in between which allowed the subjects to come from a meditative state to awake state. The order of the practice was as follows: structural scan—resting state (RS)—YN/body scan—RS—rhythmic breathing—RS—YN/body scan—RS. The YN and body scan orders were counterbalanced across subjects to negate order effects. For this paper, we focused on the analysis of the YN and RS practices. The analysis of body scan and rhythmic breathing and their comparison with YN will be part of future study. Participants followed the audio-guided YN practice in an eyes-closed supine position for 20 min, the transcript is provided in Fig. 1 and the audio (in English in the voice of Sri Sri Ravi Shankar, founder Art of Living) is publicly available^91^.

### Data acquisition

We acquired our data on a Philips Ingenia 3.0 T whole-body scanner at Mahajan Imaging, Gurugram. We used a T1-weighted 3D MP-RAGE protocol (FOV = 250 × 250 mm, flip angle = 8°, acquisition voxel size = 1 × 1 mm, slice thickness = 1 mm, 250 slices, slice gap =  − 0.40 mm) to collect high-resolution anatomical data. The blood oxygen level-dependent (BOLD) functional data during YN paradigm was acquired using the multi-band echo-planar imaging pulse sequence (TR = 1000 ms, TE = 25 ms, flip angle = 65°, FOV = 219 × 219 mm, acquisition voxel size = 3 × 3 mm, slice thickness = 4 mm, 48 slices without slice gap). The initial 26 subjects were acquired with a multi-band factor of eight and the remaining with the factor three. The functional data during resting states for all subjects had TR = 1000 ms, TE = 25 ms, 65° flip angle, FOV = 219 × 219 mm, voxel size = 3 × 3 × 4 mm, 60 slices without slice gap, and a multi-band factor of four.

### General linear model of YN paradigm

#### Preprocessing

High-resolution T1-weighted were preprocessed using FSL’s BET (frac = 0.3) to remove non-brain tissues. Subsequent preprocessing of functional MRI (fMRI) data involved FSL’s MCFLIRT for motion correction and temporal high-pass filtering (cut-off: 100 s). The slice-time correction and spatial smoothing using a Gaussian kernel with a 5 mm full-width at half-maximum (FWHM) was applied. Images were then registered using FSL’s FLIRT with subject-specific anatomical images (7-DOF) and the MNI152 standard space (12-DOF)^92^.

#### First level general linear model (GLM)

Analysis was applied to the pre-processed fMRI data using FSL’s FEAT (FMRI expert analysis tool). The explanatory variable (EV) in the model was the presence of audio instructions, coded as one for the duration of an instruction (refer to Fig. 1 for detailed timing of the instructions). This was done with the aim of modeling the BOLD response in relation to the audio instruction. The model was convolved with the canonical hemodynamic response function (HRF), and temporal filtering was applied to match the filtering performed during preprocessing.

#### Identifying subjects not following YN instructions

From the first level GLM analysis based on auditory instruction presence during yoga nidra, we obtained an FDR-corrected Z-statistic image for each subject. We calculated the number of significant voxels in auditory association and language comprehension ROIs from the Harvard–Oxford cortical Atlas (superior temporal gyrus, anterior and posterior divisions; middle temporal gyrus, anterior and posterior divisions; and planum temporale). The primary auditory area (Heschl’s gyri) was excluded to avoid activation solely from the audio instruction or scanner noise without a corresponding activation in comprehension areas. Subjects with no active voxels in the aforementioned ROIs were excluded, possibly indicating that they either slept during yoga nidra or couldn’t hear the instructions due to scanner noise. This resulted in the exclusion of two out of 30 meditators and seven out of 31 controls. All subsequent analysis are done on finally shortlisted, 28 meditators and 24 control subjects.

#### Group level statistics

Following the first-level analysis performed for each subject, the resulting contrasts of parameter estimates (COPEs) were used to obtain group-level statistics. Employing Nilearn’s ‘SecondLevelModel’ function in Python (i) a one-sample *t*-test on all 52 subjects was used to assess whether the group-level effect significantly differs from zero (ii) an unpaired, two-sample *t*-test was applied to investigate the contrast difference between meditators (n = 28) and controls (n = 24). The resulting z-statistic maps from the second-level analyses were corrected for multiple comparisons using the false discovery rate (FDR) method at an alpha level of 0.05, and voxels surviving FDR correction are reported and considered statistically significant in the context of our study.

#### Functional connectivity analysis

Functional connectivity (FC) analysis was performed for 28 meditators and 24 controls during both YN and resting state paradigms. For the resting state paradigm, data from three meditators and one control were unavailable.

#### Preprocessing

Preprocessing of fMRI data for both YN and resting state paradigms involved FSL’s MCFLIRT for motion correction, BET brain extraction, temporal high-pass filtering (cut-off: 100 s) and slice-timing corrections. The FMRIB automated segmentation tool (FAST) from FSL was employed to extract the cerebrospinal fluid (CSF) tissues from the T1-weighted images^92^. The generated CSF mask was then intersected with a standard ventricle ROI. A noise vector (csf_ts) was calculated from the average signal of the CSF × ventricle mask. Head motion parameters—three rotational (x, y, z in radians) and three translational (x, y, z in millimeters) for each volume—were derived from the MCFLIRT motion correction outputs. These six head-motion parameters, along with the CSF signal, constituted seven noise vectors that spanned the noise subspace. The component orthogonal to the noise vectors (i.e., perpendicular to the noise basis) was employed for subsequent analyses. In the YN FC analysis, we incorporated two additional confounds as noise regressors to refine our model. These included the hemodynamic response function (HRF) convolved with the timing and duration of audio instructions (as described in GLM analysis) and the first derivative of this convolved signal. For both YN and resting state, we employed a stringent motion correction protocol for FC analysis. Frame-wise displacement (FD) was calculated based on the six motion parameters, and any frames exhibiting a displacement greater than 0.5 mm were censored^93^. Preprocessed and de-noised fMRI data were then subjected to spatial smoothing using a Gaussian kernel with a 5 mm FWHM and registered using FSL’s FLIRT with subject-specific anatomical images (7-DOF) to the MNI152 standard space (12-DOF)^92^.

#### FC calculation

A priori default mode network (DMN) seeds—the posterior cingulate cortex (PCC) [MNI:(0, − 53,26)], medial prefrontal cortex (mPFC) [MNI:(0,52, − 6)], left inferior parietal lobule (left-IPL)[MNI:(− 48, − 62,36)], and right inferior parietal lobule (right-IPL) [MNI:(46, − 62,36)]—were employed in the FC analysis^62^. Time series from a sphere of radius 10 mm centered on these coordinates were extracted, and pairwise correlations with all other voxels in the brain were computed. For the resting state scans 4 min time series was selected (t_start_ = 30 s, t_end_ = 270 s). The 20 min long YN scan, was divided into four periods: T1 (t_start_ = 0 s, t_end_ = 240 s), T2 (t_start_ = 240 s, t_end_ = 480 s), T3 (t_start_ = 530 s, t_end_ = 770 s) each lasting 240 s, and T4 (t_start_ = 830 s, t_end_ = 1045 s) spanning 215 s for connectivity analysis. These periods were chosen so that they exclude any explicit breathing instructions and are each matching with the duration of the RS to the best possible extent. As a result the duration of T1, T2 and T3 match exactly with RS and T4 is 25 s shorter. After standardizing these time series data using their z-score, we created a correlation matrix depicting the FC between each DMN seed and all other voxels in the brain. These correlation matrices underwent Fisher-Z transformation to facilitate group-level statistical analyses. For ROI analysis, the definitions of the three subnetworks of default network—A, B and C were extracted from the Yeo 17 network atlas^94^ and illustrated in Fig. S10. One-sample *t*-test was performed on all subjects (n = 52) to obtain group statistics. The two-sample *t*-test was conducted on each matrix element to determine significant differences in FC between groups.

#### ANOVA

We conducted a two-way ANOVA to assess the FC of the DMN seed ROIs (PCC, mPFC, right-IPL and left-IPL) with the Default A network, as defined by the Schaefer cortical Atlas. The ANOVA model included two factors: group (meditators vs. controls) and stage (RS_PRE, YN_T1, YN_T2, YN_T3, YN_T4, RS_POS). Additionally, we examined the interaction effect between group and stage.

#### Relationship between total meditation practice and DMN-FC

Scatter plots were created to examine the relationship between the FC of DMN seeds to the Default A network and self-reported cumulative practice hours (yoga asana, pranayama, and meditation) for all subjects. Spearman’s rank test was performed to obtain p-values, which were FDR-corrected for multiple comparisons (see Table S1).

### Ethical consideration

The study was reviewed and approved by the Institute Ethics Committee (IEC) of the Indian Institute of Technology, Delhi on 22/09/2017 (Ethics application: P-014) as prescribed by the Ethical guidelines for biomedical research on human participants of ICMR (Indian Council of Medical Research).

### Supplementary Information

Supplementary Information.

## Data Availability

The datasets used and/or analysed during the current study available from the corresponding author on reasonable request.
