# Supplementary material for: Functional connectivity changes in meditators and novices during yoga nidra practice
Source: Sci Rep. 2024 Jun 5;14:12957. doi: 10.1038/s41598-024-63765-7 (PMC11153538; doi:10.1038/s41598-024-63765-7)
Supplement: Supplementary file 1 — Supplementary Information. [file 41598_2024_63765_MOESM1_ESM.pdf]

Supplementary Information for

Functional Connectivity Changes in Meditators and Novices during Yoga Nidra Practice

Suruchi Fialoke[1a], Vaibhav Tripathi[1b], Sonika Thakral[c], Anju Dhawan[d], Vidur Majahan[e], Rahul Garg*[a,f,g]

***To whom correspondence should be addressed, email: rahulgarg@cse.iitd.ac.in**
[1]Contributed equally to the work

[a]*National Resource Center for Value Education in Engineering, Indian Institute of Technology, Delhi, India*
[b]*Psychological and Brain Sciences, Boston University, Boston, USA*
[c]*Department of Computer Science, Shaheed Sukhdev College of Business Studies, University of Delhi*
[d]*National Drug Dependence Treatment Centre, All India Institute of Medical Sciences (AIIMS), Delhi, India,*
[e]*Mahajan Imaging, Delhi, India*
[f]*Amar Nath and Shashi Khosla School of Information Technology, Indian Institute of Technology, Delhi, India*
[g]*Department of Computer Science and Engineering, Indian Institute of Technology, Delhi, India*

Keywords: Yoga-nidra, fMRI, Default Mode Network, meditation, functional connectivity

This PDF file includes:

Supplementary Figures S1 to S12
Supplementary Tables S1 to S7

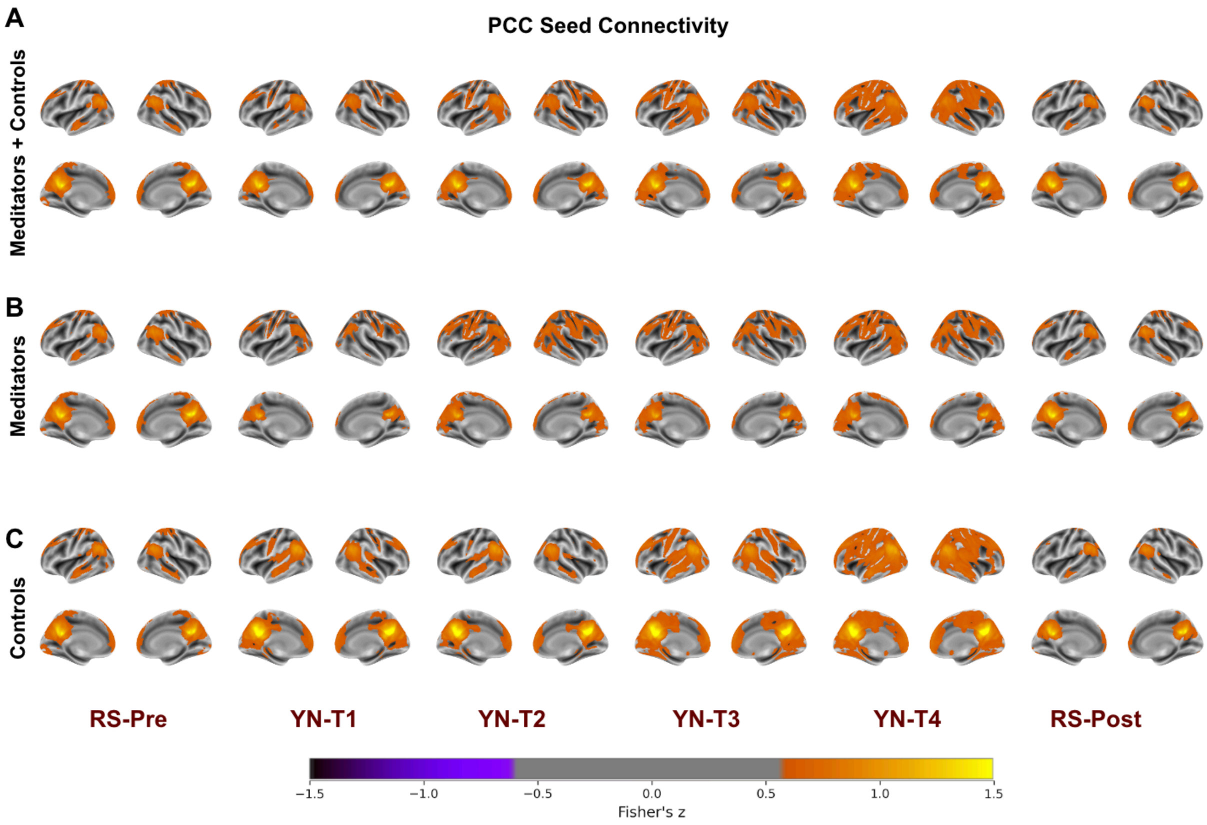

Figure S1. Functional Connectivity (FC) maps during Resting States (RS) and Yoga Nidra Practice employing apriori DMN seed, Posterior Cingulate Cortex (PCC) [MNI:(0,-53,26)]. The intensity of FC is displayed on surface maps using Fisher's z-value of Pearson's correlation, thresholded at z=0.6. The color scale represents the strength of the correlation, with warmer colors indicating stronger connectivity.

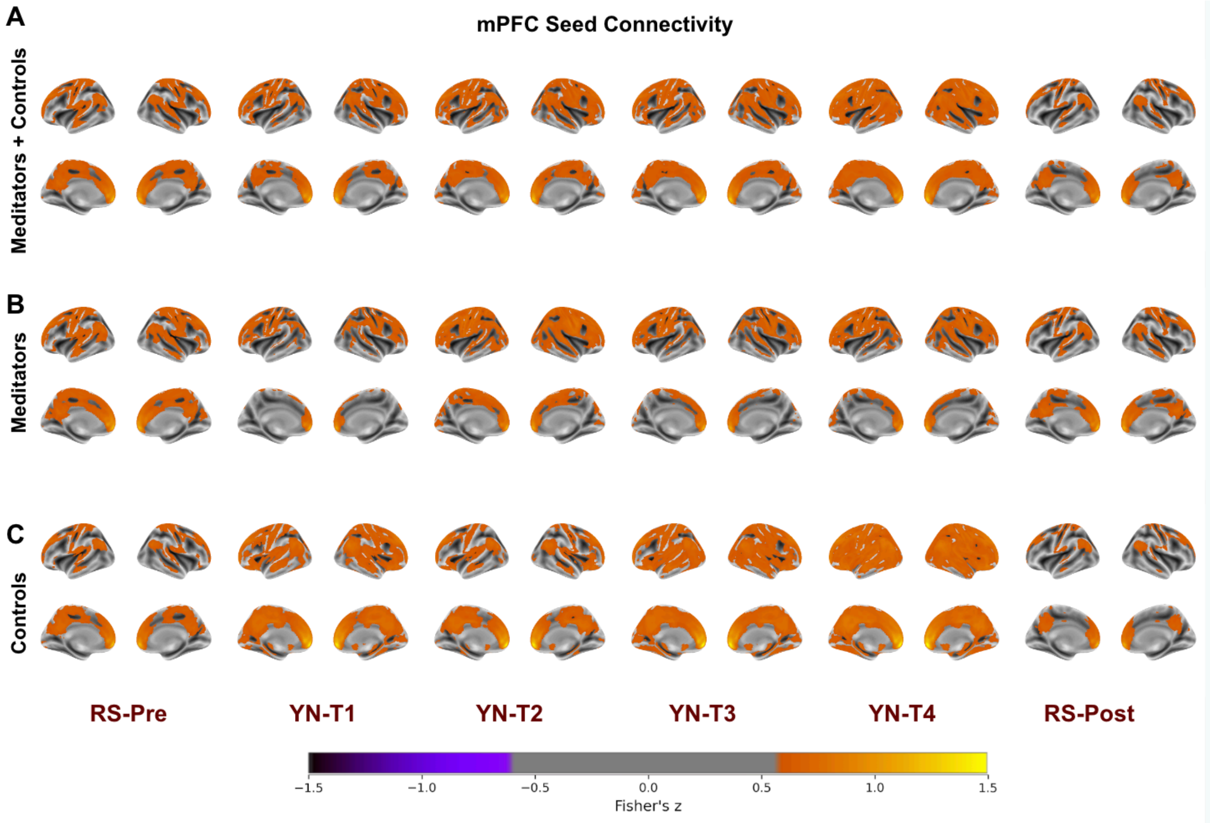

Figure S2. Functional Connectivity (FC) maps during Resting States (RS) and Yoga Nidra Practice employing medial Prefrontal Cortex (mPFC) seed [MNI:(0,52,-6)]. The intensity of FC is displayed on surface maps using Fisher's z-value of Pearson's correlation, thresholded at z=0.6. The color scale represents the strength of the correlation, with warmer colors indicating stronger connectivity.

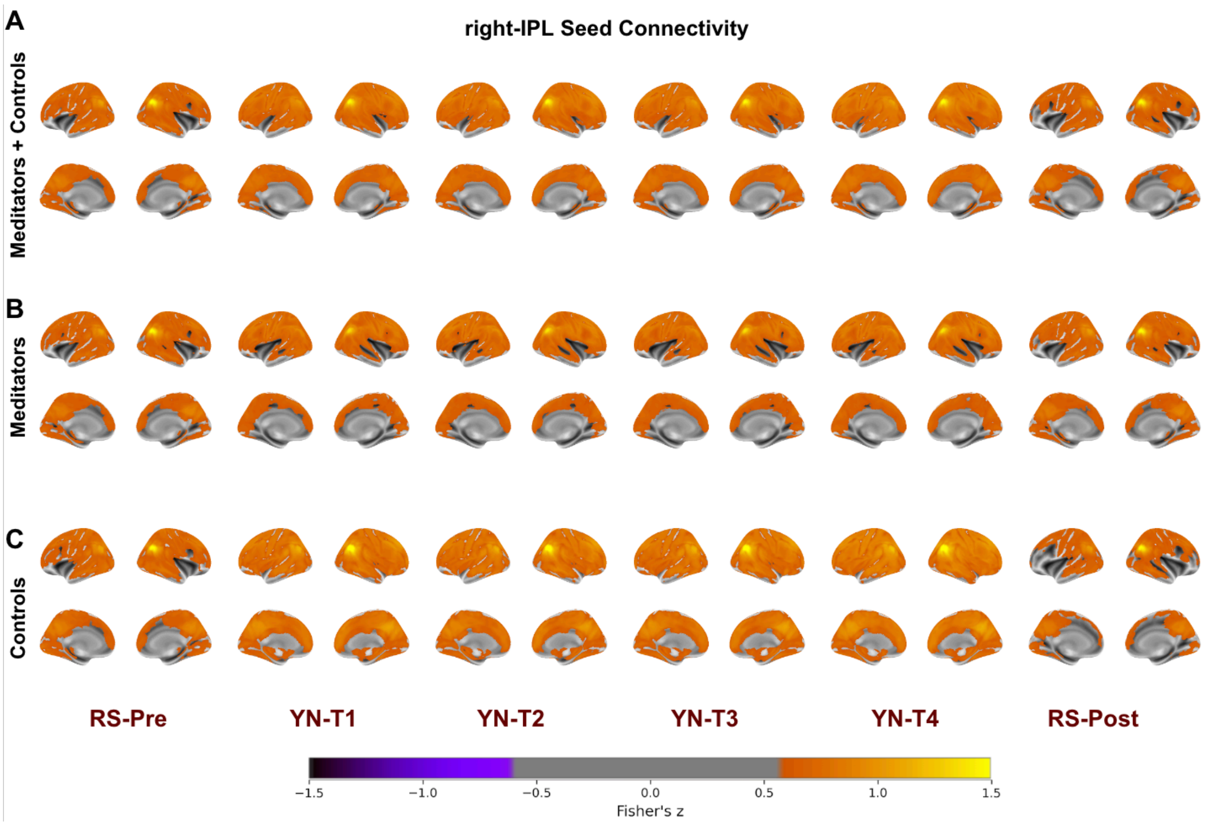

Figure S3. Functional Connectivity (FC) maps during Resting States (RS) and Yoga Nidra Practice employing right Inferior Parietal Lobule (right-IPL) seed [MNI:(46, -62, 36)]. The intensity of FC is displayed on surface maps using Fisher's z-value of Pearson's correlation, thresholded at z=0.6. The color scale represents the strength of the correlation, with warmer colors indicating stronger connectivity.

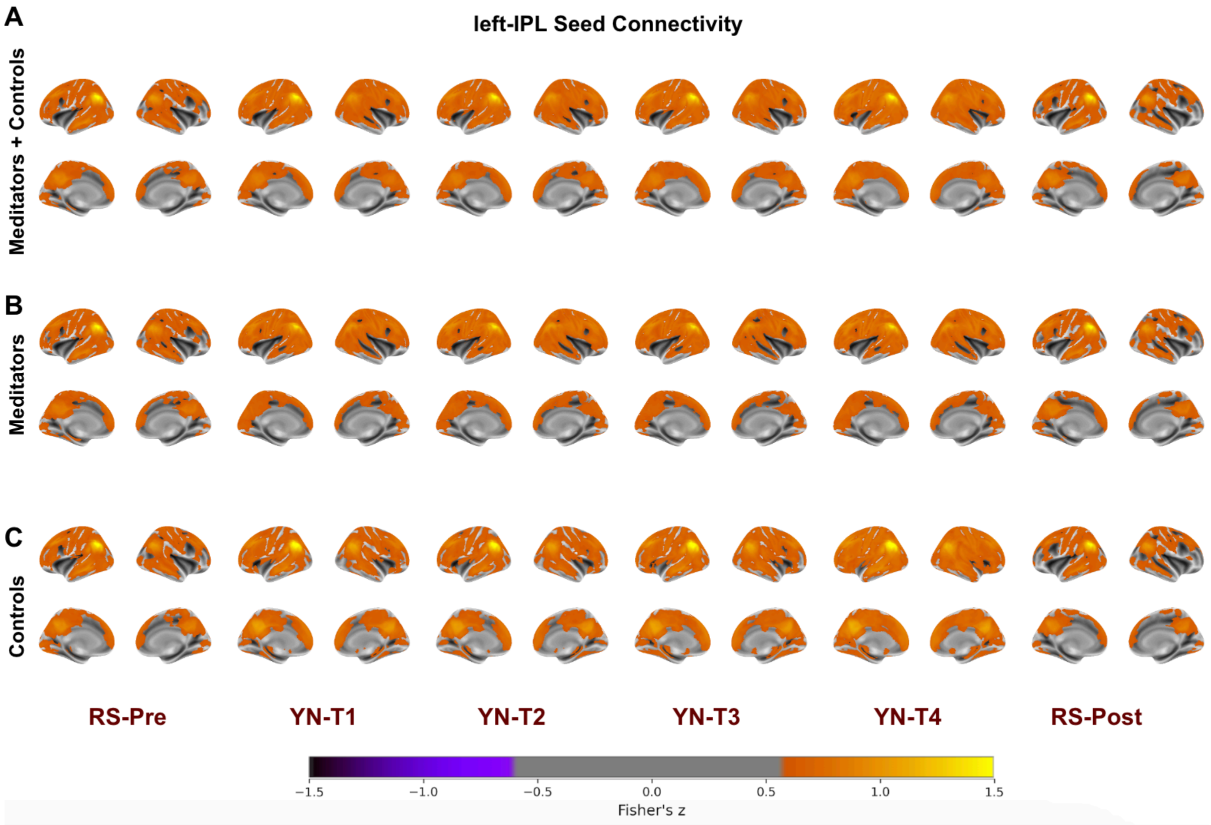

Figure S4. Functional Connectivity (FC) maps during Resting States (RS) and Yoga Nidra Practice employing left Inferior Parietal Lobule (left-IPL) seed [MNI:(-48, -62, 36)]. The intensity of FC is displayed on surface maps using Fisher's z-value of Pearson's correlation, thresholded at z=0.6. The color scale represents the strength of the correlation, with warmer colors indicating stronger connectivity.

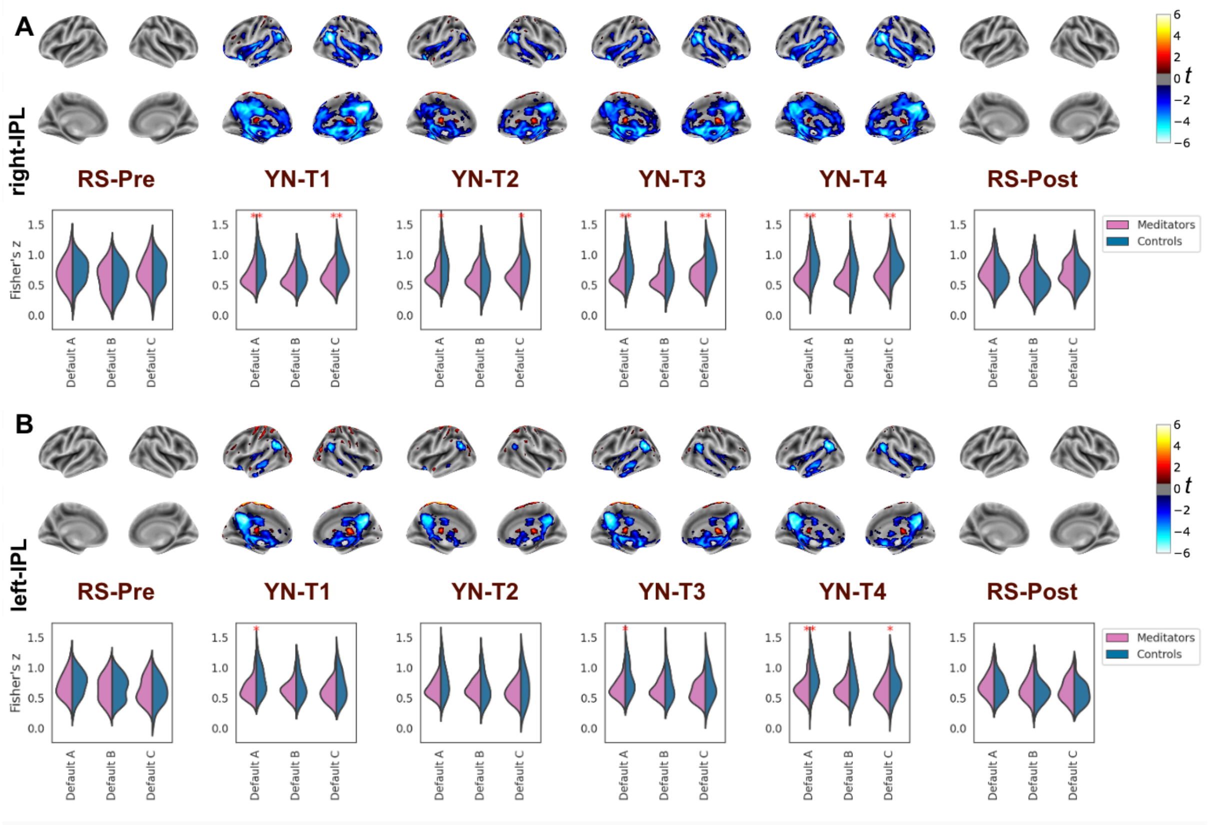

Fig. S5. Group Differences in DMN-FC between meditators and controls during Yoga Nidra but not during resting states. The figure uses two-sided t-tests to compare FC in meditators and controls using DMN seeds (A) right-IPL and (B) left-IPL. The surface plots display the t-values, corrected for multiple comparisons (FDR-corrected, q < 0.05), across the four stages of Yoga Nidra (YN), as well as in resting states pre and post-YN. The accompanying violin plots illustrate the distribution of Fisher's z-values for both groups during these stages. These plots represent the averaged Fisher's z-values of the seeds within three distinct Default Mode Network (DMN) subdivisions (Default A, Default B, and Default C), as outlined by the Schaefer Cortical Atlas. The width of the violin plot at any given y-value (Fisher's z-value) represents the proportion of data located there, providing a visual representation of the data's distribution.

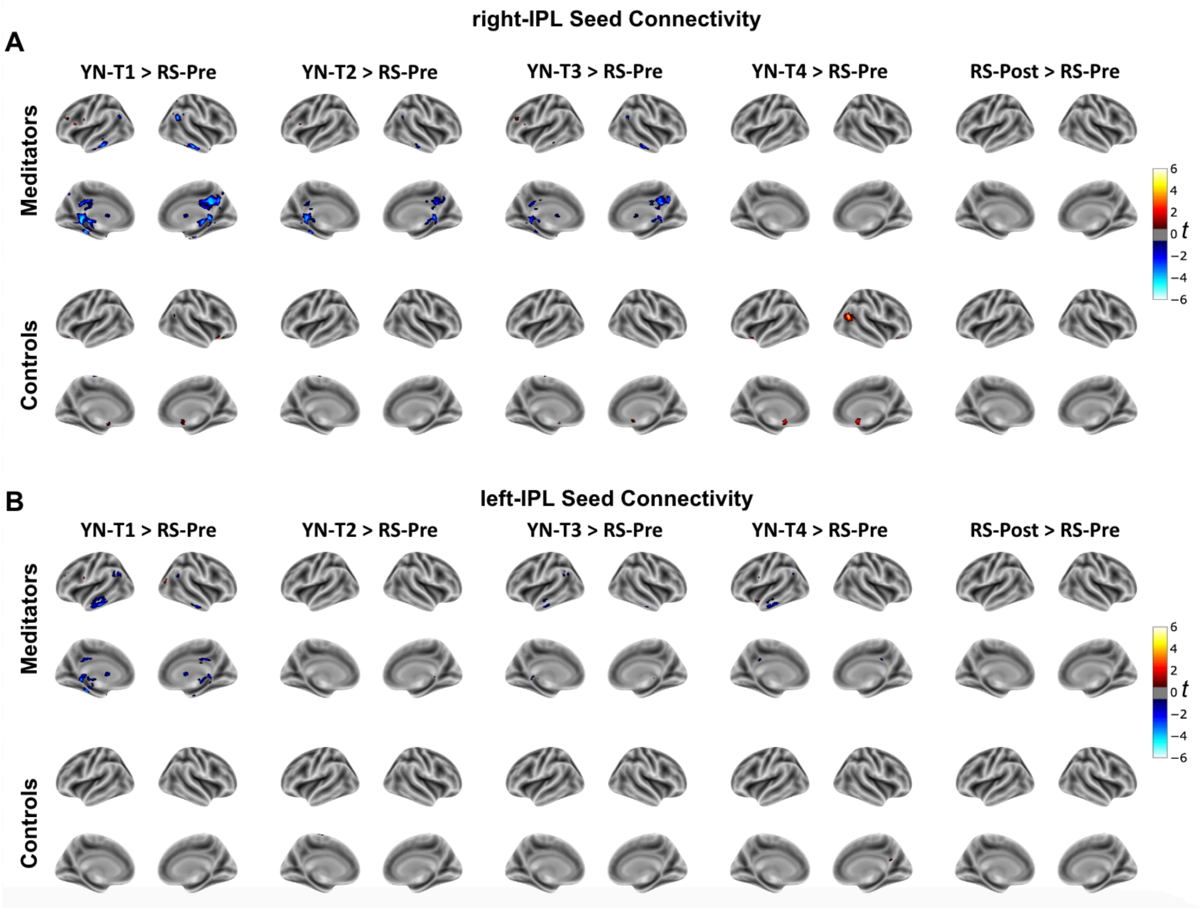

Figure S6. Functional connectivity changes in meditators and controls during Yoga Nidra compared to rest. For (A) right-IPL and (B) left-IPL seeds, a comparison of the FC during YN (T1 through T4) and the resting state post-completion of Yoga Nidra (RS-Post) is performed with resting state pre-Yoga Nidra (RS-Pre) as a baseline. The surface maps present t-values from two-sided t-tests, corrected for multiple comparisons (FDR-corrected, q < 0.05). The figure highlights that meditators demonstrate a significant decrease in DMN connectivity during YN compared to their resting state. Conversely, controls display a slight increase or no change in connectivity during YN relative to RS-Pre.

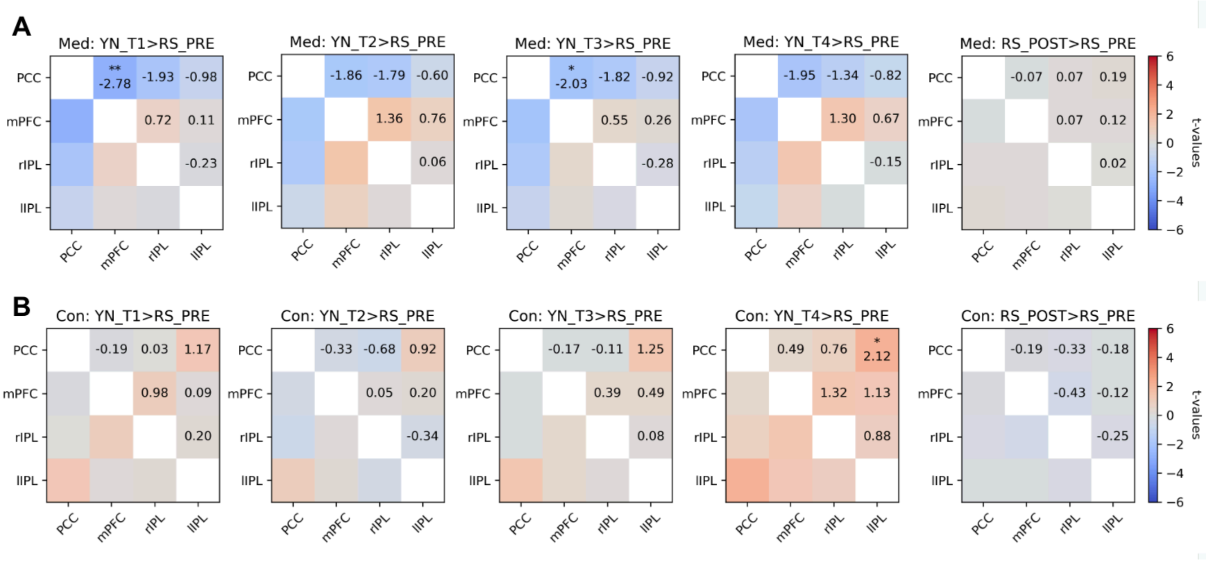

Figure S7: For (A) Meditators and (B) Controls, the comparison of intra-DMN FC (between DMN nodes) of stages of YN and RS-Post, using RS-Pre as the reference baseline. Results of the t-test between the Fisher's z values of FC among DMN-node pairs for each stage and RS-Pre are presented. The color bars in the figure correspond to the associated t-values.

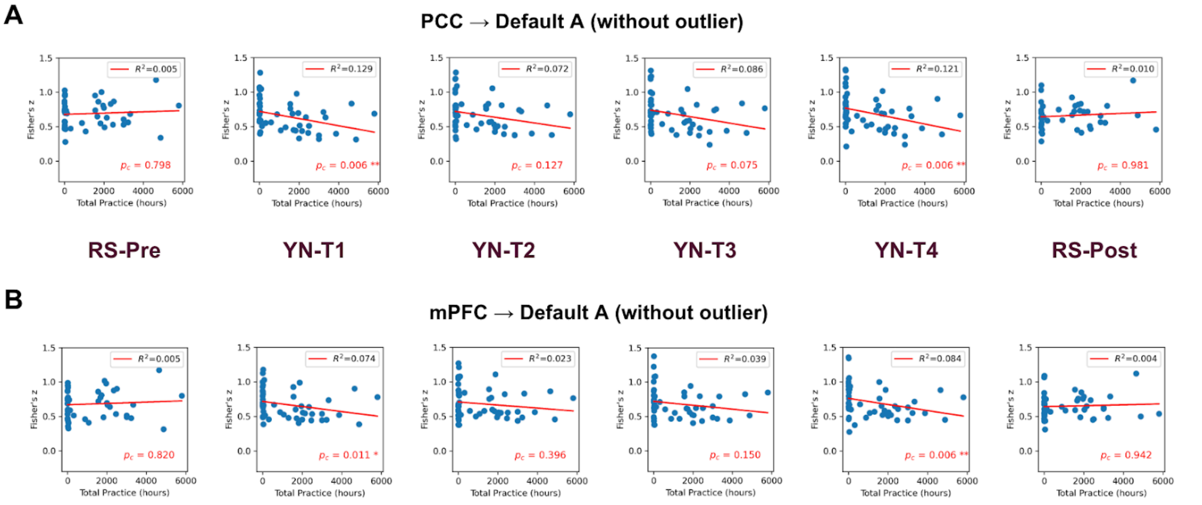

Figure S8. Correlation between the total duration of meditation practice and the functional connectivity (FC) of (A) mPFC seed and (B) PCC seed with other regions of the DMN (Default A as defined by the Schaefer Atlas) during the Yoga Nidra (YN) practice. This analysis replicates Figure 5 from the main text, with the exception of the outlier practitioner whose cumulative practice hours exceeded 8000.

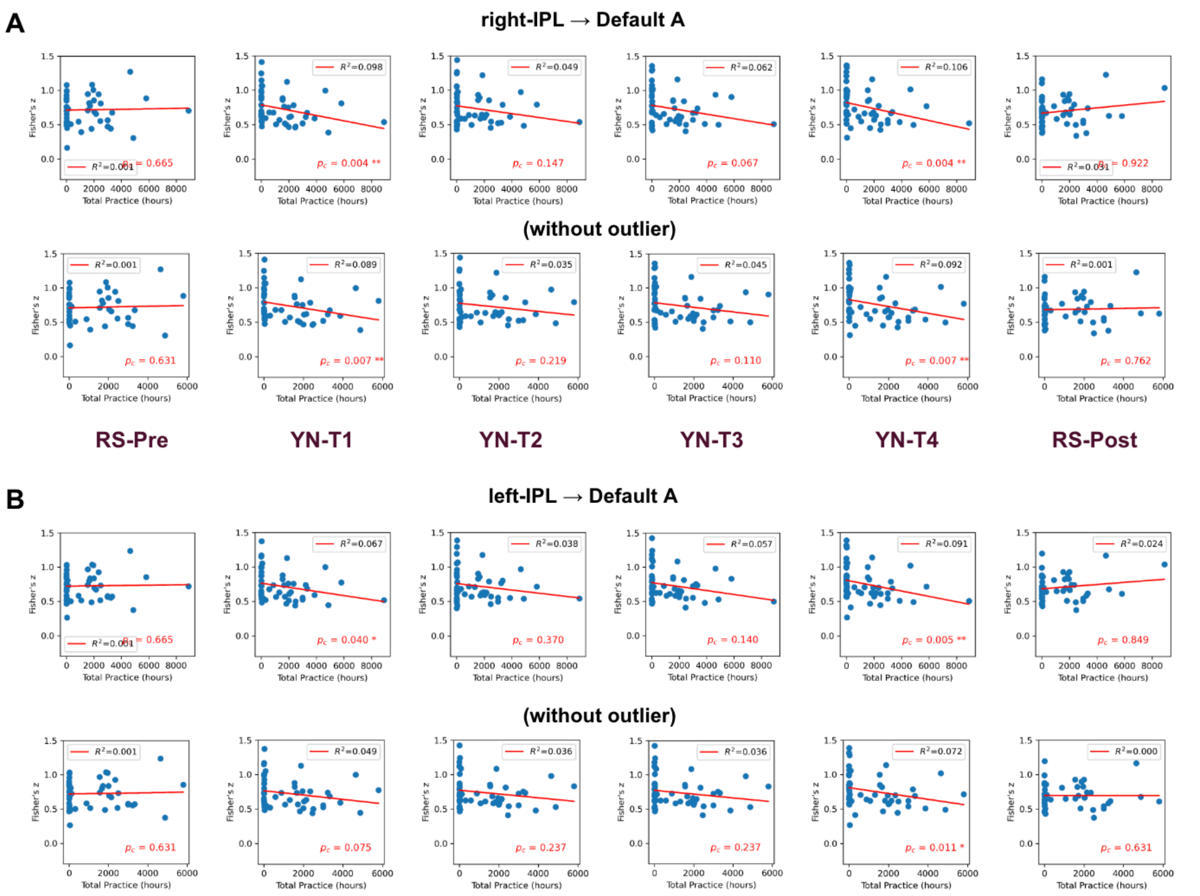

Figure S9. Correlation between the total duration of meditation practice and the functional connectivity (FC) of (A) right-IPL seed and (B) left-IPL seed with other regions of the DMN (Default A as defined by the Schaefer Atlas) during the Yoga Nidra (YN) practice. Sub-plots "without outliers" are generated after removing the practitioner whose cumulative practice hours exceeded 8000.

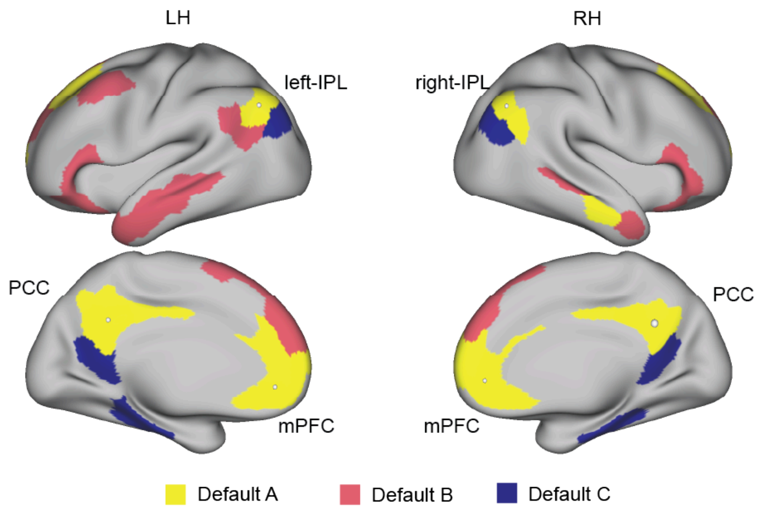

Figure S10. Default Network and ROI definitions

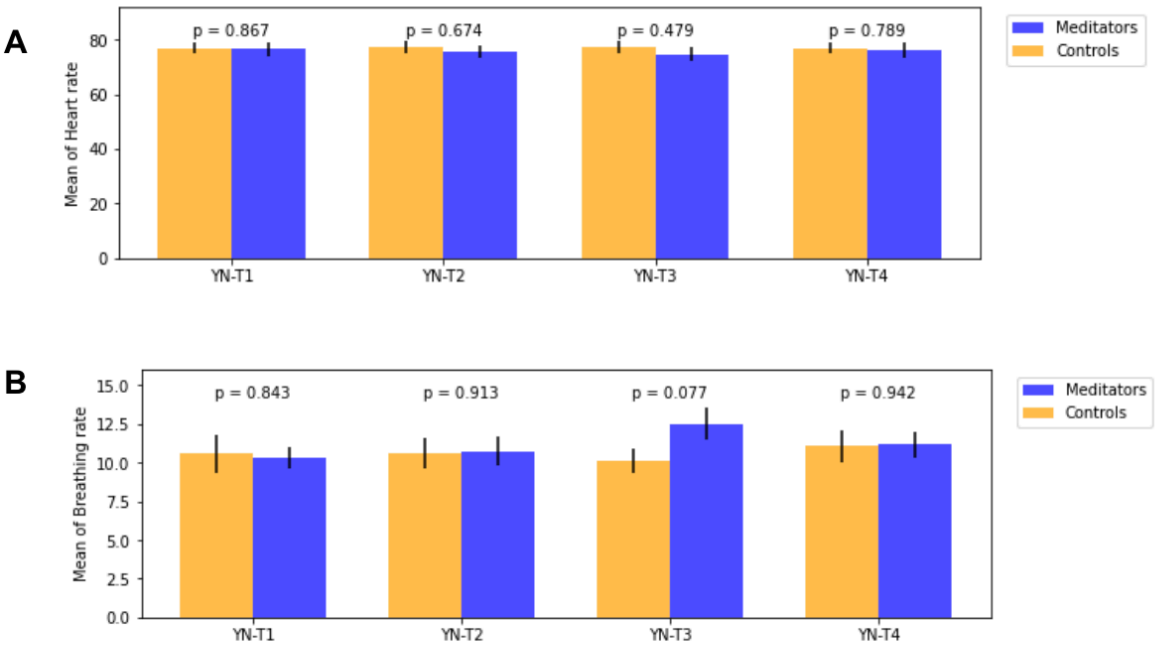

Figure S11: Results of t-test of (A) mean heart rate (per minute) and (B) mean breathing rate (per minute) between Meditators (n=19) and Controls (n=17). No significant difference was found in any stage of YN. The heart rate and breathing rates were obtained from ECG data (obtained during fMRI) using the Python Library HeartPy[1].

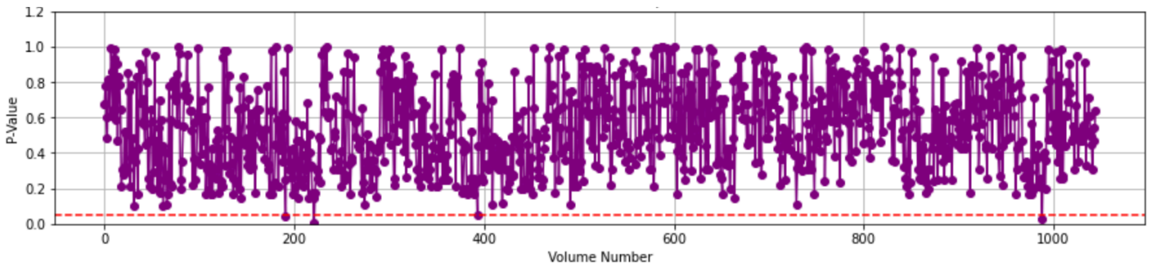

Figure S12: Plot illustrating frame-wise p-values (FDR corrected) comparing the mean Framewise Displacement (FD) of meditators versus controls. The red dotted line corresponds to p=0.05. Only 4 out of 1145 (0.3%) frames had significant differences between the motion of meditators versus controls.

Table S1: Parameters from scatter plots between **total meditation practice and DMN-FC between various DMN Seeds to Default A.**

			Pearson correlation coefficient	Spearman rank correlation test		
**Seed**	**Paradigm**	**DOF**	**Rval**	**SP_Coef**	**SP_Pval**	**SP_Pval_FDR**
**PCC**	**YN-T1**	50	-0.352	-0.468	0.000	0.004
**PCC**	**YN-T2**	50	-0.276	-0.284	0.041	0.082
**PCC**	**YN-T3**	50	-0.307	-0.333	0.016	0.043
**PCC**	**YN-T4**	50	-0.350	-0.503	0.000	0.003
**PCC**	**RS-PRE**	46	-0.027	-0.088	0.553	0.699
**PCC**	**RS-POST**	46	0.186	0.053	0.719	0.822
**mPFC**	**YN-T1**	50	-0.345	-0.441	0.001	0.004
**mPFC**	**YN-T2**	50	-0.224	-0.202	0.150	0.241
**mPFC**	**YN-T3**	50	-0.282	-0.294	0.035	0.075
**mPFC**	**YN-T4**	50	-0.341	-0.488	0.000	0.003
**mPFC**	**RS-PRE**	46	-0.005	-0.068	0.647	0.776
**mPFC**	**RS-POST**	46	0.178	0.035	0.814	0.849
**right-IPL**	**YN-T1**	50	-0.313	-0.450	0.001	0.004
**right-IPL**	**YN-T2**	50	-0.220	-0.240	0.086	0.147
**right-IPL**	**YN-T3**	50	-0.249	-0.305	0.028	0.067
**right-IPL**	**YN-T4**	50	-0.326	-0.457	0.001	0.004
**right-IPL**	**RS-PRE**	46	0.026	-0.106	0.474	0.665
**right-IPL**	**RS-POST**	46	0.176	-0.015	0.922	0.922
**left-IPL**	**YN-T1**	50	-0.258	-0.341	0.013	0.040
**left-IPL**	**YN-T2**	50	-0.194	-0.163	0.247	0.370
**left-IPL**	**YN-T3**	50	-0.239	-0.248	0.076	0.140
**left-IPL**	**YN-T4**	50	-0.302	-0.431	0.001	0.005
**left-IPL**	**RS-PRE**	46	0.024	-0.100	0.499	0.665
**left-IPL**	**RS-POST**	46	0.156	-0.039	0.794	0.849

Table S2. GLM analysis of Yoga Nidra, all subjects (meditators and controls collectively) revealed the following significant clusters sorted by the z-statistic value at the peak. The MNI coordinate of the peak z-stat value and corresponding ROI names from the Harvard Oxford atlas are provided.

Cluster-ID	X	Y	Z	Peak Stat	Cluster (mm3)	ROI Name
1	60	8	-10	9.76	420640	Temporal Pole, Superior Temporal Gyrus, anterior division
1a	-64	-20	6	8.97		Superior Temporal Gyrus, posterior division, Planum Temporale, Heschl's Gyrus (includes H1 and H2),
1b	64	-18	2	8.87		Superior Temporal Gyrus, posterior division, Planum Temporale
1c	-60	0	-4	8.80		Superior Temporal Gyrus, anterior division
2	-26	2	-10	4.02	1016	Left Putamen
3	-18	18	-14	3.55	472	Frontal Orbital Cortex
4	8	-44	-52	3.39	488	Brain-Stem
5	-10	-30	40	3.16	360	Cingulate Gyrus, posterior division, Precentral Gyrus
6	-22	-22	14	2.95	232	Left Thalamus
7	-22	-38	46	2.87	224	Postcentral Gyrus
1	-46	-62	26	-4.36	3424	Lateral Occipital Cortex, superior division, Angular Gyrus
2	-8	34	54	-3.81	1656	Superior Frontal Gyrus
2a	-16	52	44	-3.06		Frontal Pole
3	-56	4	-36	-3.80	1624	Temporal Pole
4	14	62	28	-3.76	1272	Frontal Pole
5	52	4	-42	-3.69	1128	Inferior Temporal Gyrus, anterior division, Temporal Pole
6	18	32	58	-3.44	560	Superior Frontal Gyrus
7	-66	-16	-26	-3.43	312	Middle Temporal Gyrus, posterior division
8	24	-74	20	-3.41	1816	Lateral Occipital Cortex, superior division
8a	42	-78	12	-3.14		Lateral Occipital Cortex, inferior division, Lateral Occipital Cortex, superior division
8b	22	-82	34	-2.85		Lateral Occipital Cortex, superior division
8c	36	-78	12	-2.81		Lateral Occipital Cortex, inferior division, Lateral Occipital Cortex, superior division
9	-6	-50	36	-3.40	408	Precuneous Cortex, Cingulate Gyrus, posterior division
10	46	-58	24	-3.40	968	Angular Gyrus, Lateral Occipital Cortex, superior division
11	28	-48	-6	-3.24	304	Lingual Gyrus, Temporal Occipital Fusiform Cortex
12	-32	-84	12	-3.03	520	Lateral Occipital Cortex, superior division
13	38	-66	18	-2.95	176	Lateral Occipital Cortex, superior division
14	-12	62	32	-2.92	224	Frontal Pole
15	-16	-82	20	-2.78	176	Lateral Occipital Cortex, superior division

Table S3. PCC Seed FC difference between meditators and Controls, results of t-test within three distinct Default Mode Network (DMN) subdivisions (Default A, Default B, and Default C), as outlined by the Schaefer Cortical Atlas.

Stage	Network	Mean Expert	Mean Control	StdErr Expert	StdErr Control	T-Stat	P-Value
RS_PRE	DefaultA	0.686	0.683	0.040	0.042	0.048	0.9619
RS_PRE	DefaultB	0.531	0.542	0.044	0.043	-0.173	0.8635
RS_PRE	DefaultC	0.661	0.636	0.038	0.042	0.431	0.6689
YN_T1	DefaultA	0.557	0.766	0.033	0.043	-3.942	0.0003
YN_T1	DefaultB	0.488	0.565	0.031	0.041	-1.524	0.1337
YN_T1	DefaultC	0.541	0.731	0.034	0.046	-3.379	0.0014
YN_T2	DefaultA	0.592	0.751	0.035	0.053	-2.577	0.0130
YN_T2	DefaultB	0.532	0.547	0.033	0.055	-0.256	0.7991
YN_T2	DefaultC	0.577	0.703	0.036	0.054	-1.994	0.0516
YN_T3	DefaultA	0.583	0.791	0.035	0.052	-3.406	0.0013
YN_T3	DefaultB	0.511	0.607	0.031	0.054	-1.618	0.1120
YN_T3	DefaultC	0.555	0.753	0.033	0.052	-3.322	0.0017
YN_T4	DefaultA	0.588	0.818	0.035	0.053	-3.698	0.0005
YN_T4	DefaultB	0.520	0.651	0.031	0.056	-2.118	0.0392
YN_T4	DefaultC	0.581	0.794	0.036	0.052	-3.446	0.0012
RS_POS	DefaultA	0.682	0.639	0.035	0.039	0.825	0.4135
RS_POS	DefaultB	0.511	0.498	0.040	0.039	0.229	0.8200
RS_POS	DefaultC	0.645	0.602	0.033	0.037	0.870	0.3890

Table S4. mPFC Seed FC difference between meditators and Controls, results of t-test within three distinct Default Mode Network (DMN) subdivisions (Default A, Default B, and Default C), as outlined by the Schaefer Cortical Atlas.

**Stage**	**Network**	**Mean Expert**	**Mean Control**	**StdErr Expert**	**StdErr Control**	**T-Stat**	**P-Value**
RS_PRE	DefaultA	0.685	0.671	0.043	0.043	0.218	0.8282
RS_PRE	DefaultB	0.601	0.580	0.044	0.047	0.325	0.7467
RS_PRE	DefaultC	0.579	0.559	0.044	0.042	0.329	0.7436
YN_T1	DefaultA	0.581	0.763	0.032	0.042	-3.505	0.0010
YN_T1	DefaultB	0.538	0.638	0.034	0.040	-1.897	0.0636
YN_T1	DefaultC	0.494	0.646	0.032	0.041	-2.940	0.0050
YN_T2	DefaultA	0.626	0.735	0.033	0.054	-1.789	0.0796
YN_T2	DefaultB	0.594	0.607	0.034	0.052	-0.215	0.8307
YN_T2	DefaultC	0.534	0.607	0.030	0.051	-1.274	0.2086
YN_T3	DefaultA	0.602	0.767	0.031	0.051	-2.872	0.0060
YN_T3	DefaultB	0.557	0.656	0.030	0.052	-1.704	0.0945
YN_T3	DefaultC	0.507	0.649	0.029	0.049	-2.584	0.0127
YN_T4	DefaultA	0.604	0.813	0.029	0.053	-3.620	0.0007
YN_T4	DefaultB	0.566	0.705	0.029	0.057	-2.288	0.0264
YN_T4	DefaultC	0.526	0.693	0.028	0.052	-2.955	0.0048
RS_POS	DefaultA	0.671	0.636	0.033	0.040	0.692	0.4922
RS_POS	DefaultB	0.568	0.535	0.036	0.045	0.566	0.5742
RS_POS	DefaultC	0.565	0.533	0.035	0.035	0.653	0.5172

Table S5. right-IPL Seed FC difference between meditators and Controls, results of t-test within three distinct Default Mode Network (DMN) subdivisions (Default A, Default B, and Default C), as outlined by the Schaefer Cortical Atlas

Stage	Network	Mean Expert	Mean Control	StdErr Expert	StdErr Control	T-Stat	P-Value
RS_PRE	DefaultA	0.710	0.722	0.047	0.047	-0.175	0.8618
RS_PRE	DefaultB	0.609	0.619	0.049	0.047	-0.148	0.8830
RS_PRE	DefaultC	0.728	0.709	0.047	0.048	0.289	0.7739
YN_T1	DefaultA	0.644	0.840	0.033	0.049	-3.413	0.0013
YN_T1	DefaultB	0.614	0.699	0.029	0.043	-1.686	0.0981
YN_T1	DefaultC	0.650	0.825	0.034	0.046	-3.130	0.0029
YN_T2	DefaultA	0.668	0.812	0.034	0.056	-2.282	0.0268
YN_T2	DefaultB	0.642	0.675	0.032	0.053	-0.547	0.5868
YN_T2	DefaultC	0.655	0.809	0.033	0.055	-2.489	0.0162
YN_T3	DefaultA	0.654	0.837	0.032	0.056	-2.940	0.0050
YN_T3	DefaultB	0.618	0.704	0.030	0.052	-1.465	0.1491
YN_T3	DefaultC	0.661	0.834	0.032	0.050	-2.995	0.0043
YN_T4	DefaultA	0.664	0.874	0.034	0.054	-3.378	0.0014
YN_T4	DefaultB	0.629	0.748	0.028	0.055	-2.015	0.0493
YN_T4	DefaultC	0.674	0.871	0.035	0.047	-3.411	0.0013
RS_POS	DefaultA	0.708	0.676	0.041	0.044	0.530	0.5988
RS_POS	DefaultB	0.597	0.563	0.042	0.044	0.553	0.5830
RS_POS	DefaultC	0.720	0.677	0.039	0.041	0.754	0.4545

Table S6. left-IPL Seed FC difference between meditators and Controls, results of t-test within three distinct Default Mode Network (DMN) subdivisions (Default A, Default B, and Default C), as outlined by the Schaefer Cortical Atlas

Stage	Network	Mean Expert	Mean Control	StdErr Expert	StdErr Control	T-Stat	P-Value
RS_PRE	DefaultA	0.720	0.730	0.042	0.043	-0.161	0.8732
RS_PRE	DefaultB	0.656	0.667	0.044	0.042	-0.184	0.8546
RS_PRE	DefaultC	0.640	0.600	0.043	0.044	0.656	0.5150
YN_T1	DefaultA	0.655	0.802	0.031	0.049	-2.630	0.0113
YN_T1	DefaultB	0.634	0.690	0.027	0.045	-1.110	0.2725
YN_T1	DefaultC	0.609	0.698	0.032	0.049	-1.562	0.1246
YN_T2	DefaultA	0.676	0.785	0.031	0.055	-1.797	0.0784
YN_T2	DefaultB	0.659	0.673	0.029	0.049	-0.255	0.7997
YN_T2	DefaultC	0.615	0.683	0.032	0.057	-1.085	0.2833
YN_T3	DefaultA	0.664	0.818	0.030	0.053	-2.627	0.0114
YN_T3	DefaultB	0.644	0.719	0.027	0.052	-1.334	0.1883
YN_T3	DefaultC	0.606	0.715	0.030	0.053	-1.869	0.0674
YN_T4	DefaultA	0.669	0.851	0.033	0.054	-2.970	0.0046
YN_T4	DefaultB	0.652	0.749	0.028	0.054	-1.676	0.1000
YN_T4	DefaultC	0.625	0.761	0.034	0.053	-2.227	0.0305
RS_POS	DefaultA	0.712	0.694	0.037	0.040	0.341	0.7346
RS_POS	DefaultB	0.643	0.622	0.038	0.040	0.381	0.7053
RS_POS	DefaultC	0.633	0.583	0.037	0.038	0.938	0.3531

Table S7: 2-Way ANOVA Results on FC of DMN ROIs within Default A Network (as outlined by the Schaefer Cortical Atlas) by Groups (Meditators, Controls) and Stages (RS_PRE, YN_T1, YN_T2, YN_T3, YN_T4, RS_POS).

PCC -> Default A				
	sum_sq	df	F	PR(>F)
C(Group)	1.285	1	30.005	9.308E-08
C(Stage)	0.061	5	0.287	9.203E-01
C(Group):C(Stage)	0.868	5	4.054	1.425E-03
Residual	12.501	292		

mPFC -> Default A				
	sum_sq	df	F	PR(>F)
C(Group)	0.850	1	20.889	7.193E-06
C(Stage)	0.064	5	0.314	9.042E-01
C(Group):C(Stage)	0.665	5	3.270	6.875E-03
Residual	11.876	292		

right-IPL -> Default A				
	sum_sq	df	F	PR(>F)
C(Group)	1.128	1	23.403	2.130E-06
C(Stage)	0.145	5	0.599	7.004E-01
C(Group):C(Stage)	0.654	5	2.711	2.057E-02
Residual	14.079	292		

left-IPL -> Default A				
	sum_sq	df	F	PR(>F)
C(Group)	0.756	1	17.419	3.961E-05
C(Stage)	0.072	5	0.331	8.942E-01
C(Group):C(Stage)	0.422	5	1.948	8.648E-02
Residual	12.669	292		

References

[1] van Gent, P., Farah, H., van Nes, N., & van Arem, B. (2018). Heart Rate Analysis for Human Factors: Development and Validation of an Open Source Toolkit for Noisy Naturalistic Heart Rate Data. In Proceedings of the 6th HUMANIST Conference (pp. 173–178).
